# Long noncoding RNA *Regulating ImMune Escape* regulates mixed lineage leukaemia protein‐1‐H3K4me3‐mediated immune escape in oesophageal squamous cell carcinoma

**DOI:** 10.1002/ctm2.1410

**Published:** 2023-09-15

**Authors:** Jia Liu, Wei‐Yi Zhou, Xiao‐Jing Luo, Yan‐Xing Chen, Chau‐Wei Wong, Ze‐Xian Liu, Jia‐ Bo Zheng, Hai‐ Yu Mo, Jun‐Quan Chen, Jia‐Jun Li, Ming Zhong, Yu‐Hong Xu, Qi‐Hua Zhang, Heng‐Ying Pu, Qi‐Nian Wu, Ying Jin, Zi‐Xian Wang, Rui‐Hua Xu, Hui‐Yan Luo

**Affiliations:** ^1^ Department of Medical Oncology Sun Yat‐sen University Cancer Center State Key Laboratory of Oncology in South China Collaborative Innovation Center for Cancer Medicine Sun Yat‐sen University Guangzhou P. R. China; ^2^ Research Unit of Precision Diagnosis and Treatment for Gastrointestinal Cancer Chinese Academy of Medical Sciences Guangzhou P. R. China

**Keywords:** esophageal squamous cell carcinoma, immune escape, immunotherapy, lncRNA, mixed lineage leukaemia protein‐1 (MLL1)

## Abstract

**Background:**

Predictive biomarkers for oesophageal squamous cell carcinoma (ESCC) immunotherapy are lacking, and immunotherapy resistance remains to be addressed. The role of long noncoding RNA (lncRNA) in ESCC immune escape and immunotherapy resistance remains to be elucidated.

**Methods:**

The tumour‐associated macrophage‐upregulated lncRNAs and the exosomal lncRNAs highly expressed in ESCC immunotherapy nonresponders were identified by lncRNA sequencing and polymerase chain reaction assays. CRISPR‐Cas9 was used to explore the functional roles of the lncRNA. RNA pull‐down, MS2‐tagged RNA affinity purification (MS2‐TRAP) and RNA‐binding protein immunoprecipitation (RIP) were performed to identify lncRNA‐associated proteins and related mechanisms. In vivo, the humanized PBMC (hu‐PBMC) mouse model was established to assess the therapeutic responses of specific lncRNA inhibitors and their combination with programmed cell death protein 1 (PD‐1) monoclonal antibody (mAb). Single‐cell sequencing, flow cytometry, and multiplex fluorescent immunohistochemistry were used to analyze immune cells infiltrating the tumour microenvironment.

**Results:**

We identified a lncRNA that is involved in tumour immune evasion and immunotherapy resistance. High *LINC02096* (*RIME*) expression in plasma exosomes correlates with a reduced response to PD‐1 mAb treatment and poor prognosis. Mechanistically, *RIME* binds to mixed lineage leukaemia protein‐1 (MLL1) and prevents ankyrin repeat and SOCS box containing 2 (ASB2)‐mediated MLL1 ubiquitination, improving the stability of MLL1. *RIME*‐MLL1 increases H3K4me3 levels in the promoter regions of programmed death‐ligand 1 (PD‐L1) and indoleamine 2,3‐dioxygenase 1 (IDO‐1), constitutively increasing the expression of PD‐L1/IDO‐1 in tumour cells and inhibiting CD8^+^ T cells infiltration and activation. *RIME* depletion in huPBMC‐NOG mice significantly represses tumour development and improves the effectiveness of PD‐1 mAb treatment by activating T‐cell‐mediated antitumour immunity.

**Conclusions:**

This study reveals that the *RIME*‐MLL1‐H3K4me3 axis plays a critical role in tumour immunosuppression. Moreover, *RIME* appears to be a potential prognostic biomarker for immunotherapy and developing drugs that target *RIME* may be a new therapeutic strategy that overcomes immunotherapy resistance and benefits patients with ESCC.

## Graphical abstract

In the tumour microenvironment, exosome transmission and tumour necrosis factor‐α stimulation induce *RIME* upregulation in tumour cells. *RIME* binds to MLL1 and prevents ASB2‐mediated ubiquitination, which enhances MLL1 stability and increases the enrichment of H3K4me3 in the *PD‐L1* and *IDO‐1* promoter regions, thus upregulating tumoral expression of immune checkpoint markers PD‐L1/IDO‐1. This upregulation contributes to T‐cell dysfunction and blocks the antitumour immune response, which facilitates immune evasion and tumour development.

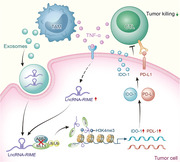

## 1 INTRODUCTION

Oesophageal squamous cell carcinoma (ESCC) is a widespread malignancy worldwide that is more prevalent in China than anywhere else. Previously, the prognosis and treatment options for ESCC patients were very poor.^1^ However, the advent of immunotherapy, such as immune checkpoint programmed cell death protein 1 (PD‐1) monoclonal antibody (mAb) immunotherapy, overcame the treatment bottleneck, and other clinical trials have explored different immunotherapy combination regimens with first‐line treatments of advanced ESCC, including chemotherapy and targeted therapy.^2^, ^3^, ^4^ Among them, the ESCORT‐1st trial conducted by our group found that the administration of a PD‐1 mAb (camrelizumab) in combination with chemotherapy significantly improved progression‐free survival and overall survival of advanced or metastatic ESCC patients.^3^ Even so, only a minority of patients benefitted from immunotherapy, the response rate and OS of ESCC patients are still far from satisfactory, and the mechanism of resistance to immunotherapy remains to be elucidated. Therefore, additional investigation on how to identify patients who will benefit from immunotherapy and how to increase immunotherapy effectiveness is required.^5^, ^6^

Exosomes are secreted by many cell types, including immune cells, adipocytes, stem cells, and cancer cells. Biomolecules inside exosomes, such as proteins and noncoding RNAs, are transferred from cell to cell, which facilitates cell‐cell communication and results in many diseases, including cancer, and resistance to cancer therapy.^7^, ^8^ Exosome‐mediated signalling in the tumour microenvironment is required for regulating innate and adaptive immune responses.^9^ As one of the most abundant contents in exosomes, exosomal lncRNAs are key messenger molecules in the local environment. Exosomal lncRNAs are reported to mediate immune evasion and influence the response to immunotherapy, but the mechanism remains largely unknown.^10^ For example, exosomal lncRNA ENST00000560647 inhibits dendritic cell‐mediated antigen presentation, inactivates CD8^+^ T cells and promotes escape from immune surveillance.^11^ In addition, exosomal lncRNAs can be detected in biological fluids such as blood and urine,^8^ which is advantageous as a potential predictor of tumour immunotherapy response.

Tumour immune evasion is a hallmark of cancer, as well as one of the most important causes of the progression of ESCC.^12^ An in‐depth exploration of the mechanism of immune escape can aid the development of precision diagnosis and treatment of ESCC patients and is of great importance for improving the outcomes of ESCC patients. Tumour‐associated macrophages (TAMs) are crucial in promoting tumour progression and mediating tumour immune escape. Our previous study found that a macrophage‐associated lncRNA, *MALR*, is a crucial regulator of tumour aerobic glycolysis and ESCC development by driving interleukin enhancer‐binding factor 3‐mediated liquid‐liquid phase separation and activating the hypoxia‐inducible factor 1 pathway.^13^ TAM‐associated lncRNAs are also involved in tumour proliferation, metastasis, angiogenesis, and therapy resistance by regulating various physiological processes, such as oncogenic signalling pathway transduction and activation and transcriptional and posttranscriptional regulation of oncogenic proteins.^7^, ^8^, ^14^ There is significant interest in investigating how TAM‐associated lncRNAs contribute to immunotherapy resistance and ESCC immune escape.

In this study, we identified that *LINC02096* is associated with the outcomes of ESCC immunotherapy. We gave it the acronym *RIME*, which stands for LncRNA‐Regulating Immune Evasion. The *RIME* high expression group had a poorer response to PD‐1 mAb treatment and a worse prognosis. Mechanistically, *RIME* bound and stabilized histone‐lysine N‐methyltransferase Mixed Lineage Leukaemia Protein‐1 (MLL1) and prevented Ankyrin Repeat and SOCS Box Containing 2 (ASB2)‐mediated MLL1 ubiquitination.^15^ *RIME*‐MLL1 epigenetically upregulated the expression of immunosuppressive molecules PD‐L1/IDO‐1 in tumour cells and caused T‐cell dysfunction and immune evasion by promoting the accumulation of H3K4me3 in their promoter regions. This study suggests that *RIME* is a potential predictive biomarker to distinguish patients who would benefit from PD‐1 mAb immunotherapy. *RIME* inhibition in combination with PD‐1 mAb may be an effective combination regimen for ESCC therapy.

## 2 RESULTS

### 2.1 LncRNA *RIME* is upregulated in ESCC patient plasma exosomes

**FIGURE 1:**
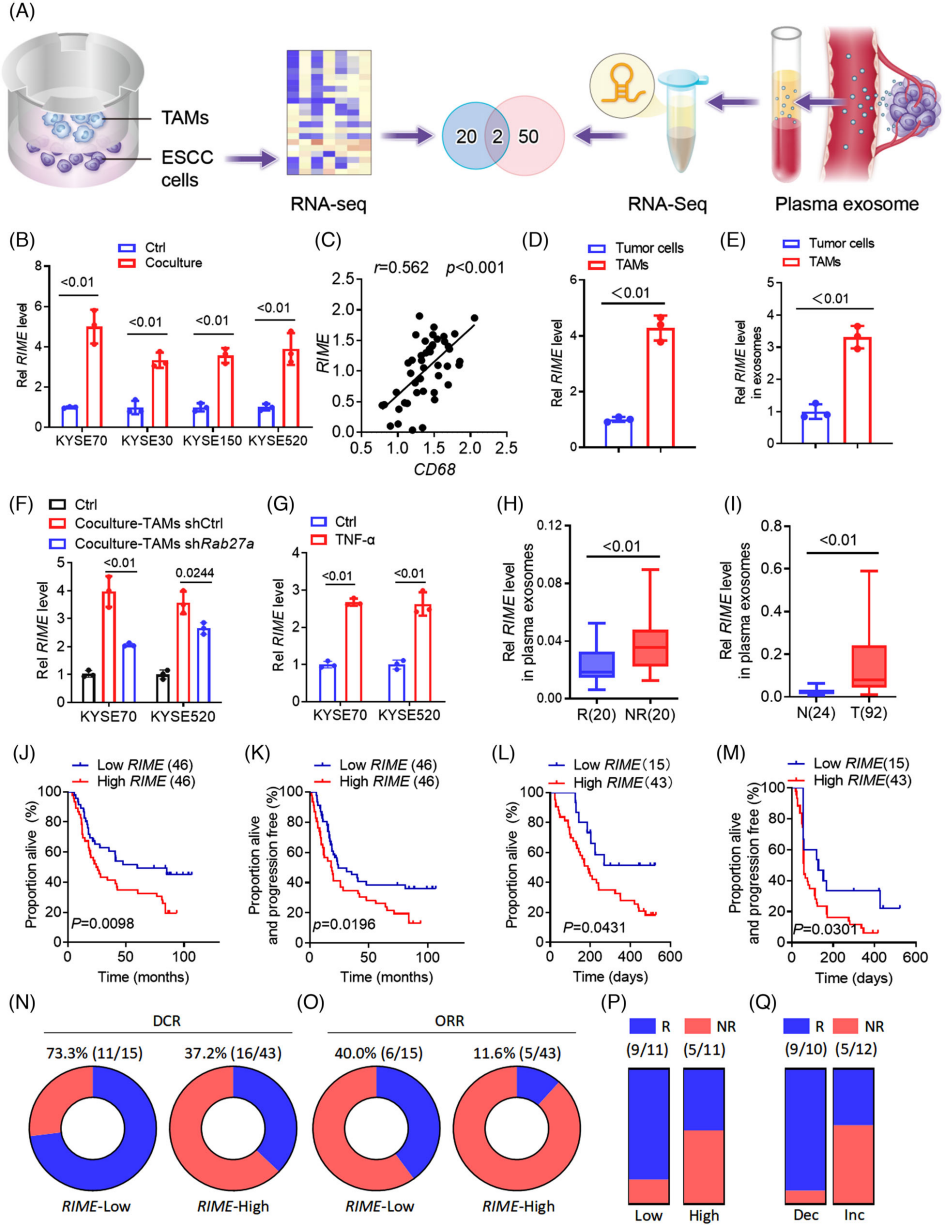
LncRNA *RIME* correlates with immunotherapy outcomes. (A) Illustration of tumour‐associated macrophage (TAM)‐upregulated long noncoding RNAs (lncRNAs) and plasma exosomal lncRNAs upregulated in oesophageal squamous cell carcinoma (ESCC) immunotherapy nonresponders. (B) quantitative real‐time polymerase chain reaction (qRT‒PCR) analysis showed that *RIME* expression was upregulated in ESCC cells cocultured with TAMs from ESCC patients. Ctrl, control. (C) qRT‒PCR and Pearson correlation analysis showed that *RIME* and *CD68* expression in ESCC tumour tissues was positively correlated. (D) qRT‐PCR analysis showed that *RIME* was more highly expressed in TAMs than in ESCC tumour cells. (E) qRT‒PCR analysis of *RIME* expression in exosomes isolated from ESCC cells and TAMs, which showed that *RIME* was more highly expressed in exosomes from TAMs. (F) qRT‒PCR analysis showed that *RIME* expression was only partially suppressed in ESCC cells co‐cultured with Rab27a‐depleted TAMs. (G) qRT‒PCR analysis showed that TNF‐α stimulation upregulated *RIME* expression in ESCC cells. (H) qRT‒PCR analysis showed that *RIME* expression was increased in the plasma exosomes of ESCC PD‐1 mAb non‐responders compared to responders. R, responders. NR, non‐responders. (I) qRT‒PCR analysis showed that *RIME* expression was increased in the plasma exosomes of ESCC patients compared to healthy donors. T, ESCC patients. N, healthy donors. (J, K) OS and PFS analysis showed that high *RIME* expression in plasma correlated with poor OS and PFS of ESCC patients. (L, M) OS and PFS analysis showed that high *RIME* expression in plasma shortened OS and PFS of ESCC patients treated with PD‐1 mAb. (N, O) The disease control rate (DCR) and objective response rate (ORR) of PD‐1 mAb treatment was lower in the *RIME* high expression group. (P) The response rate of PD‐1 mAb plus chemotherapy (TP) in the plasma *RIME* high expression group was lower than *RIME* low expression group. (Q) The response rate of PD‐1 mAb plus chemotherapy (TP) in the plasma *RIME* increased (Inc) or decreased (Dec) group. Plasma *RIME* levels were examined before and after PD‐1 mAb plus chemotherapy (TP) treatment. qRT‐PCR analysis showed that the response rate was lower in the *RIME*‐increased group.

It has been reported that TAMs are crucial in promoting tumour progression and mediating tumour immune escape. To identify TAM‐associated lncRNAs, we cocultured ESCC cells and TAMs isolated from ESCC tumour tissues, and after that ESCC cells were subjected to RNA‐sequencing analysis^13^(Figure S1A). To screen for lncRNAs in plasma exosomes that are associated with immunotherapy (PD‐1 mAb) outcomes, lncRNA sequencing was carried out on samples from 3 nonresponders and 3 responders patients undergoing treatment in Sun Yat‐sen University Cancer Center (SYSUCC) (Figure S1B–D). Sequencing results identified the 20 lncRNAs most upregulated by TAMs and the top 50 lncRNAs highly expressed in ESCC immunotherapy nonresponders, and two lncRNAs were identified after their intersection (Figure 1A). In our subsequent analysis, one of the two lncRNAs was verified to be closely related to TAMs and, more importantly, to immunotherapeutic responses. We renamed this lncRNA (LINC02096) LncRNA‐**R**egulating **I**m**M**une **E**scape, or *RIME*.

By performing quantitative real‐time polymerase chain reaction (qRT‒PCR) analysis, *RIME* was confirmed to be upregulated in ESCC cells when they were cultured together with TAMs obtained from ESCC patients (Figure 1B). Additionally, a positive correlation was observed between *RIME* and *CD68* in ESCC tissues, indicating a close association between *RIME* and TAMs (Figure 1C).^8^, ^13^, ^16^ To explore the potential mechanism by which TAMs induce *RIME* upregulation, we detected *RIME* expression in TAMs, ESCC cells and exosomes from both. RIME was shown to be more abundantly expressed in TAMs than in ESCC tumour cells (Figure 1D), and *RIME* was more highly expressed in exosomes from TAMs (Figure 1E) according to our qRT‐PCR analyses. Then we knocked out *RIME* in TAMs and performed co‐culture experiments with ESCC cells. The qRT‒PCR analysis showed that the *RIME* upregulation was attenuated when cocultured with *RIME* knockout (KO) TAMs (Figure S1E). Consistently, *RIME* upregulation was also partially suppressed when ESCC cells cocultured with *Rab27a*‐depleted TAMs (Figure 1F). These data suggest that TAMs‐induced *RIME* upregulation in ESCC cells is partially due to exosome secretion.^7^ Moreover, we quantified *RIME* levels in ESCC cells that were subjected to various TAMs secreted cytokines, including interleukin (IL)‐6, tumour necrosis factor (TNF)‐α, transforming growth factor (TGF)‐β and vascular endothelial growth factor (VEGF)^16^, ^17^(Figure 1G and Figure S1F,G). We found that TNF‐α stimulation also upregulated *RIME* expression. TNF‐α is mainly secreted by macrophages and other tumour‐infiltrating immune cells, such as cytotoxic lymphocytes and natural killer (NK) cells.^18^ These data indicate that the high expression of *RIME* in tumour cells is due to TAM exosome transmission and TNF‐α stimulation in the tumour microenvironment.

### 2.2 High *RIME* expression correlates with poor immunotherapy outcomes

Then, we further confirmed that *RIME* expression was upregulated in the plasma of ESCC PD‐1 mAb nonresponders (SYSUCC) by qRT‒PCR analysis (Figure 1H). In addition, in a cohort of ESCC patients from SYSUCC, we examined whether *RIME* was increased in ESCC patients’ plasma compared to healthy donors (Figure 1I) and whether high *RIME* expression in plasma correlated with poor overall survival (OS) and progression‐free survival (PFS) (Figure 1J,K). These data highlight that *RIME* is a potential predictive marker for ESCC immunotherapy as well as a prognostic biomarker for ESCC patients.

Since *RIME* expression was upregulated in the plasma of ESCC PD‐1 mAb nonresponders, we further analysed the correlation between the *RIME* expression level and immunotherapy outcomes. qRT‒PCR analysis was performed using the plasma samples. OS and PFS were shortened in the *RIME* high expression group (Figure 1L,M), and the disease control rate (DCR) and objective response rate (ORR) of PD‐1 mAb treatment were lower in the *RIME* high expression group (DCR = 37.2% vs. 73.3%, *p* = .0194; ORR = 11.6% vs. 40.0%, *p* = .0250) (Figure 1N,O). These surprising findings suggest that *RIME* has great potential as a biomarker for ESCC PD‐1 mAb monotherapy.

Recently, a number of clinical trials have revealed that PD‐1 mAb in combination with chemotherapy significantly improves the clinical outcomes of ESCC patients.^3^, ^4^ Clinical ESCORT‐1st and JUPITER‐6 trials conducted by our group showed that PD‐1 mAb combined with paclitaxel and cisplatin (TP) significantly improved PFS and OS in patients with advanced ESCC.^3^, ^4^ Therefore, we further analysed the plasma *RIME* expression level in a cohort of ESCC patients who received PD‐1 mAb and TP combination treatment. A reduced response rate was observed in patients with higher *RIME* expression (45.5% vs. 81.8%, *p* = .1827) (Figure 1P). We also collected plasma samples from patients after 2 cycles of treatment and assessed whether *RIME* levels increased or decreased. qRT‒PCR analysis showed that lower response rates when *RIME* levels increased (41.7% vs. 90.0%, *p* = .0310) (Figure 1Q). These data further support the potential of *RIME* to distinguish patients who may benefit from immunotherapy, and monitoring *RIME* expression levels before and after treatment can help predict the outcomes of ESCC patients.

### 2.3 *RIME* inhibits the cytotoxicity of CD8^+^ T cells by regulating immune checkpoint molecules

To determine the underlying mechanism by which *RIME* affects the outcomes of immunotherapy, we analysed the correlation between *RIME* expression levels and the tumour microenvironment by bioinformatics technology. The Cancer Genome Atlas (TCGA) transcriptome data and the CIBERSORT algorithm showed a negative correlation between *RIME* expression and immune cells and tumour‐killing activity.^19^ As shown in Figure S2A, *RIME* was negatively correlated with NK cells, CD8^+^ T cells, and M1 macrophages, alongside various tumour‐suppressive cells, as well as tumour‐killing activity indicators, including PRF1, GZMA, and cytolytic activity, which suggests that *RIME* might be involved in tumour immune evasion.

**FIGURE 2:**
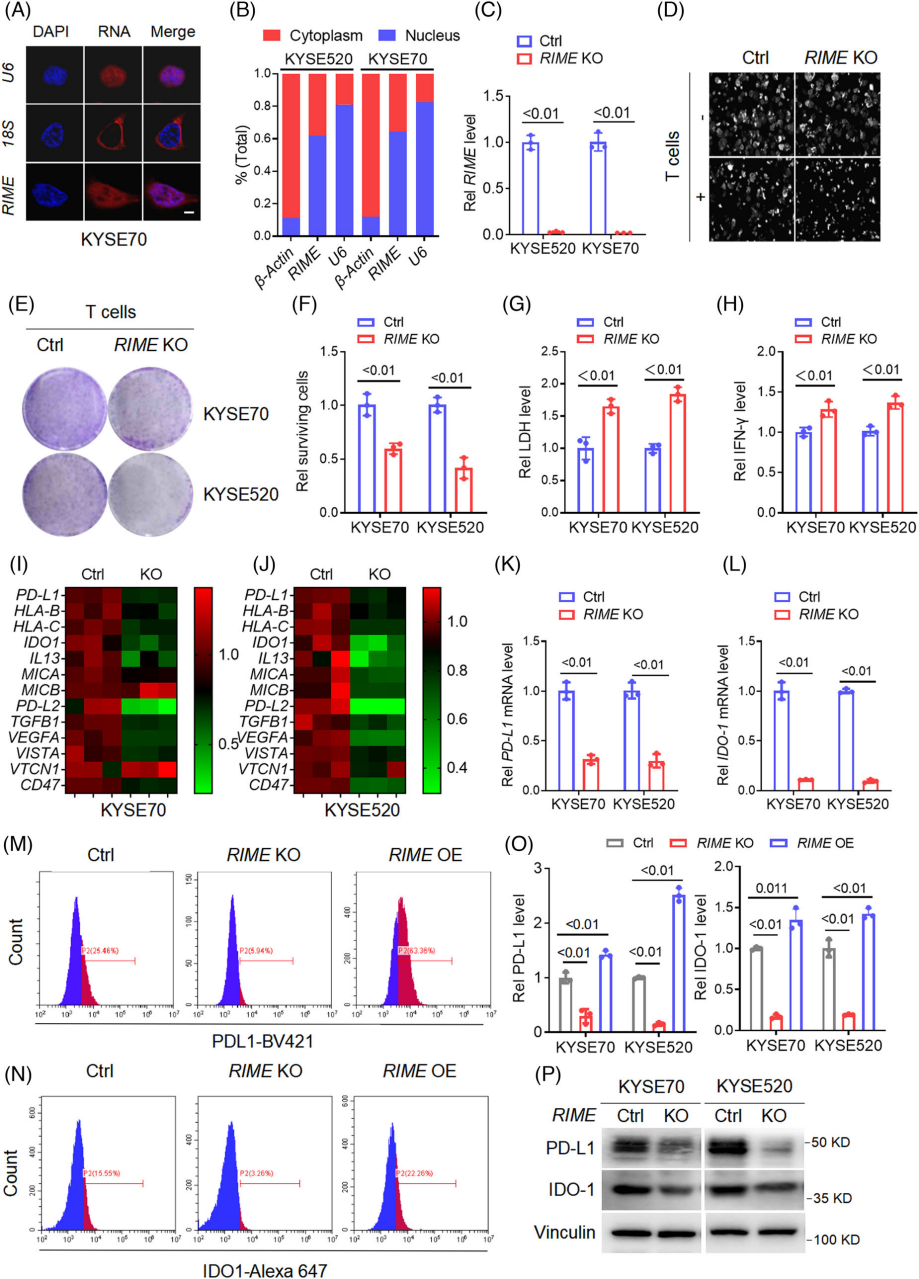
*RIME* reduces sensitivity to the cytotoxicity of CD8^+^ T cells by regulating immune checkpoint molecules. (A) Fluorescence in situ hybridization (FISH) assays showing the subcellular localization of *RIME* was both in the nucleus and cytoplasm of oesophageal squamous cell carcinoma (ESCC) cells. Scale bar, 2 μm. (B) Quantitative real‐time polymerase chain reaction (qRT‒PCR) analysis of *RIME* expression in the cytoplasmic and nuclear fractions of ESCC cells, which showed that *RIME* was localized both in the nucleus and cytoplasm. (C) qRT‒PCR analysis showed that *RIME* expression was significantly inhibited in *RIME* CRISPR KO cells. (D) Real‐time cell analyser was used to analyse the viability of ESCC cells alone or cocultured with human CD8^+^ T cells. *RIME* KO had a slight effect on ESCC cell viability but significantly enhanced the cytotoxicity of CD8^+^ T cells. (E, F) The cytotoxicity of CD8^+^ T cells to ESCC cells was determined by crystal violet staining, which showed that *RIME* KO rendered ESCC cells more sensitive to cytotoxic CD8^+^ T cells. (G) The LDH levels released from damaged cells were determined to quantify the cytotoxicity of CD8^+^ T cells. The LDH levels were significantly increased in the *RIME* KO group. (H) ELISA assays showed that the IFN‐γ levels in the coculture medium were significantly increased in the *RIME* KO group. (I, J) qRT‒PCR analysis showed that *RIME* KO significantly decreased multiple immune‐related genes in ESCC cells. (K, L) qRT‒PCR analysis showed that *RIME* KO resulted in a remarkable decrease of *PD‐L1* and *IDO‐1* expression in ESCC cells. (M–O) Flow cytometry and statistical analysis showed that *RIME* KO significantly decreased PD‐L1 and IDO‐1 expression, while *RIME* overexpression increased PD‐L1 and IDO‐1 expression. (P) Immunoblot analysis showed that *RIME* KO significantly decreased PD‐L1 and IDO‐1 protein levels in ESCC cells.

Then, we investigated the biological roles of *RIME* in vitro and in vivo. Fluorescence in situ hybridization (FISH) and cytoplasmic and nuclear fraction analysis were performed to examine the subcellular localization of *RIME*.^20^, ^21^ As shown, *RIME* was localized both in the nucleus and cytoplasm of ESCC cells (Figure 2A,B and Figure S2B). Given the subcellular localization of *RIME*, CRISPR‐Cas9 technology was used to construct *RIME* KO cells.^20^ qPCR analysis, Sanger sequencing and FISH assays confirmed that *RIME* CRISPR KO specifically and efficiently inhibited *RIME* expression in ESCC cells (Figure 2C and Figure S2C,D). Moreover, we predicted the potential off‐target locus of *RIME* sgRNA by the Cas‐Offinder tool. PCR and Sanger sequencing were performed and no off‐target loci were detected. The top 10 sequences with the highest similarity scores are listed in Table S1.

We cocultured human CD8^+^ T cells with EGFP‐labelled ESCC cells and observed the surviving ESCC cells using a real‐time cell analyser (RTCA). We found that *RIME* KO had a slight effect on ESCC cell viability while greatly increasing CD8^+^ T cell cytotoxicity (Figure 2D). A crystal violet staining assay verified that *RIME* KO increased the sensitivity of ESCC cells to cytotoxic CD8^+^ T cells (Figure 2E,F). We also measured the lactate dehydrogenase (LDH), IFN‐γ, and IL‐2 levels in the coculture medium^19^, ^22^; consistently, the levels of all of these indicators were significantly increased in the *RIME* KO group (Figure 2G,H and Figure S2E). The cocultured CD8^+^ T cells were analysed by flow cytometry and showed a significant increase in the proportion of cytotoxic CD8^+^ T cells (IFN^+^ CD8^+^ T cells) after *RIME* knockout (Figure S2F,G). Overall, *RIME* reduced sensitivity to CD8^+^ T cells cytotoxicity, which lets tumour cells evade immune surveillance and drives tumorigenesis.

To reveal the key immune response‐related genes that are affected by *RIME*, we carried out an mRNA chip analysis. *RIME* KO significantly downregulated multiple immune‐related genes, including *PD‐L1*, *IDO‐1*, *IL‐13*, and *VISTA* (Figure 2I,J).^19^, ^22^ In order to further screen for significant target genes, we constructed *RIME* OE cells and performed qPCR analysis (Figure S2H), and we found that *RIME* OE upregulated multiple immunosuppressive genes, among which *PD‐L1* and *IDO‐1* were the most significant, so we mainly focused on PD‐L1 and IDO‐1 in this study. By qPCR analysis, flow cytometric analysis, and immunoblot analysis, we further demonstrated that *RIME* KO resulted in a remarkable decrease in PD‐L1 and IDO‐1 expression levels, while *RIME* overexpression significantly increased PD‐L1 and IDO‐1 expression levels (Figure 2K–P and Figure S2I). Studies have reported the roles of PD‐L1 and IDO‐1 in inhibiting the cytotoxicity of CD8^+^ T cells and promoting immune escape. PD‐L1 in tumour cells interacts with its coinhibitory receptor PD‐1, which is found in T cells, whereby their interaction inhibits T‐cell activation, cytokine generation, and cytotoxic T lymphocyte (CTL) killer functions.^23^ IDO‐1 catalyses the oxidation of tryptophan (Trp) to produce kynurenine (Kyn), and Trp starvation and Kyn accumulation in the tumour microenvironment lead to T‐cell depletion and dysfunction.^24^ Therefore, we speculated that *RIME* might enable ESCC cells to evade antitumour immune attacks by regulating PD‐L1 and IDO‐1 expression.

### 2.4 *RIME* binds to and stabilizes MLL1

**FIGURE 3:**
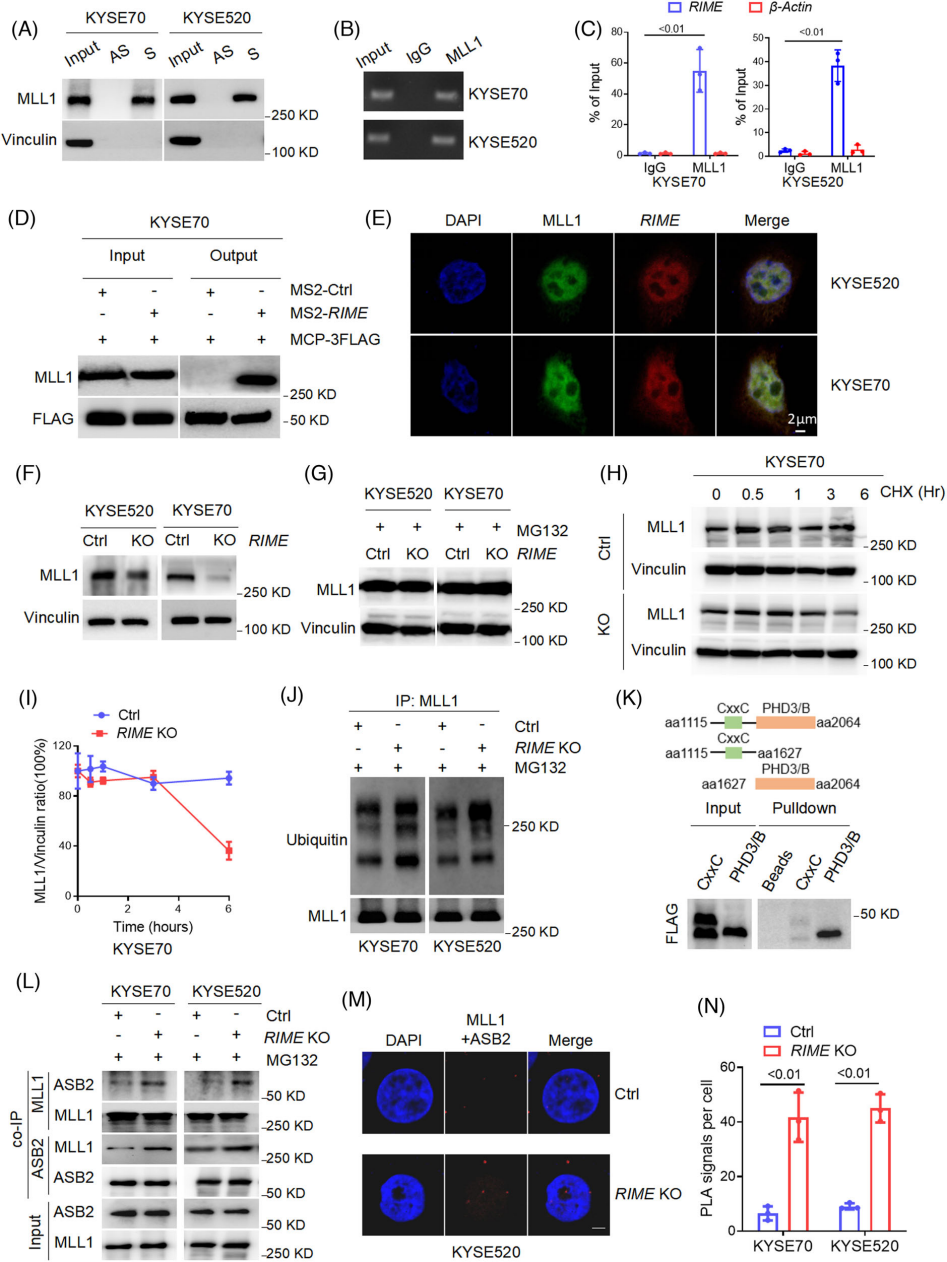
*RIME* binds and stabilizes MLL1. (A) RNA pulldown assays and immunoblot analysis showed that *RIME* specifically bound to MLL1. S, sense; AS, antisense. (B, C) RNA immunoprecipitation assays and quantitative real‐time polymerase chain reaction (qRT‒PCR) analysis verified the interaction of *RIME* and MLL1. (D) MS2‐tagged RNA affinity purification assays and immunoblot analysis verified the interaction of *RIME* and MLL1 in vivo. (E) Fluorescent in situ hybridization and immunofluorescence analysis showed that *RIME* and MLL1 colocalized mainly in the nucleus. (F) Immunoblot analysis showed that *RIME* KO remarkably reduced the MLL1 protein levels in oesophageal squamous cell carcinoma (ESCC) cells. (G) Immunoblot analysis showed that the decreased MLL1 levels in the *RIME* KO cells were recovered by the proteasomal inhibitor MG‐132 (10 μM, 12 h). (H‐I) Immunoblot analysis and quantification of MLL1 protein levels in KYSE70 cells treated with CHX (100 μg/ml) for the indicated times. As shown, *RIME* KO significantly shortened the half‐life of MLL1 in ESCC cells. (J) Immunoprecipitation and immunoblot analysis showed that *RIME* KO increased the ubiquitin levels of MLL1. (K) Schematic diagram of the MLL1 domains (top) and RNA pull‐down analysis of *RIME*‐associated FLAG‐tagged MLL1 domains (bottom). It showed that the PHD/Bromo fragment of MLL1 is required for the interaction of *RIME* and MLL1. (L) Co‐IP and immunoblot analysis showed that *RIME* KO significantly increased the MLL1/ASB2 interaction in ESCC cells. (M, N) Duolink proximity ligation assay and statistical analysis showed that *RIME* KO significantly increased the MLL1/ASB2 interaction in ESCC cells. Scale bar, 2 μm.

In order to discover the mechanism by which *RIME* controls the expression of PD‐L1 and IDO‐1, we carried out RNA pull‐down tests, and the *RIME*‐associated proteins that were eluted were analysed using mass spectrometry (Tables S2 and S3). The results of two RNA pull‐down analyses were intersected, MLL1(KMT2A) and F‐BAR and double SH3 domains protein 1 (FCHSD1) were identified as candidates. However, FCHSD1 could not be verified by immunoblot analysis, so we mainly focused on MLL1 in this study. In Figure 3A, immunoblot analysis showed that *RIME* specifically bound to MLL1.^25^ We also confirmed the specific interaction by RNA immunoprecipitation (RIP) assays and MS2‐tagged RNA affinity purification (MTRAP) assays (Figure 3B–D and Figure S3A). FISH and immunofluorescence (IF) analyses further showed that *RIME* and MLL1 colocalized mainly in the nucleus^26^ (Figure 3E).

MLL1 is a histone methyltransferase that regulates H3K4me3 and gene expression, which is indispensable in the haematopoietic process, and its upregulation leads to leukaemia and many solid tumours, such as colorectal cancer and pancreatic cancer.^25^, ^27^ To elucidate the effect of the *RIME*‐MLL1 interaction on MLL1 levels, we measured the protein level of MLL1 in *RIME* KO ESCC cells. The MLL1 level was markedly reduced in *RIME* KO ESCC cells (Figure 3F and Figure S3B). Previous studies have revealed that MLL1 is ubiquitinated and degraded by ASB2, a component of the EC S (ASB) E3 ubiquitin ligase complex.^15^ Therefore, we examined whether decreased MLL1 levels in *RIME* KO cells could be restored using the proteasomal inhibitor MG‐132. As expected, MG‐132 treatment rescued the MLL1 reduction caused by *RIME* KO (Figure 3G). *RIME* KO significantly shortened the half‐life of MLL1 in ESCC cells, suggesting that *RIME* is essential for maintaining MLL1 stability (Figure 3H,I and Figure S3C,D). Moreover, we carried out IP assays and detected the ubiquitin levels of MLL1. Consistently, *RIME* KO increased the levels of ubiquitinated MLL1 (Figure 3J and Figure S3E). These data suggest that *RIME* maintains MLL1 stability by inhibiting MLL1 ubiquitination.

### 2.5 *RIME* blocks ASB2‐mediated MLL1 degradation

It has been reported that ASB2 mediates MLL1 ubiquitination and degradation through interaction with the PHD/Bromo domain region of MLL1.^15^ To map the key domain of MLL1 that mediates its interaction with *RIME*, we performed RNA pull‐down assays and tested truncated forms of MLL1 (CxxC and PHD/Bromo domain). The PHD/Bromo fragment of MLL1 was required for this interaction in KYSE520 cells (Figure 3K). Next, we carried out co‐IP assays to investigate the effect of *RIME* on the MLL1/ASB2 interaction. As demonstrated, *RIME* KO significantly enhanced MLL1/ASB2 interaction (Figure 3L and Figure S3F), and this was further supported by the Duolink PLA assay (Figure 3 M,N). Together, these data suggest that *RIME* binds to the PHD/Bromo domain region of MLL1 and blocks ASB2‐mediated MLL1 ubiquitination and degradation, thus enhancing the stability of MLL1.

### 2.6 *RIME* regulates MLL1‐H3K4me3‐mediated PD‐L1 and IDO‐1 expression

**FIGURE 4:**
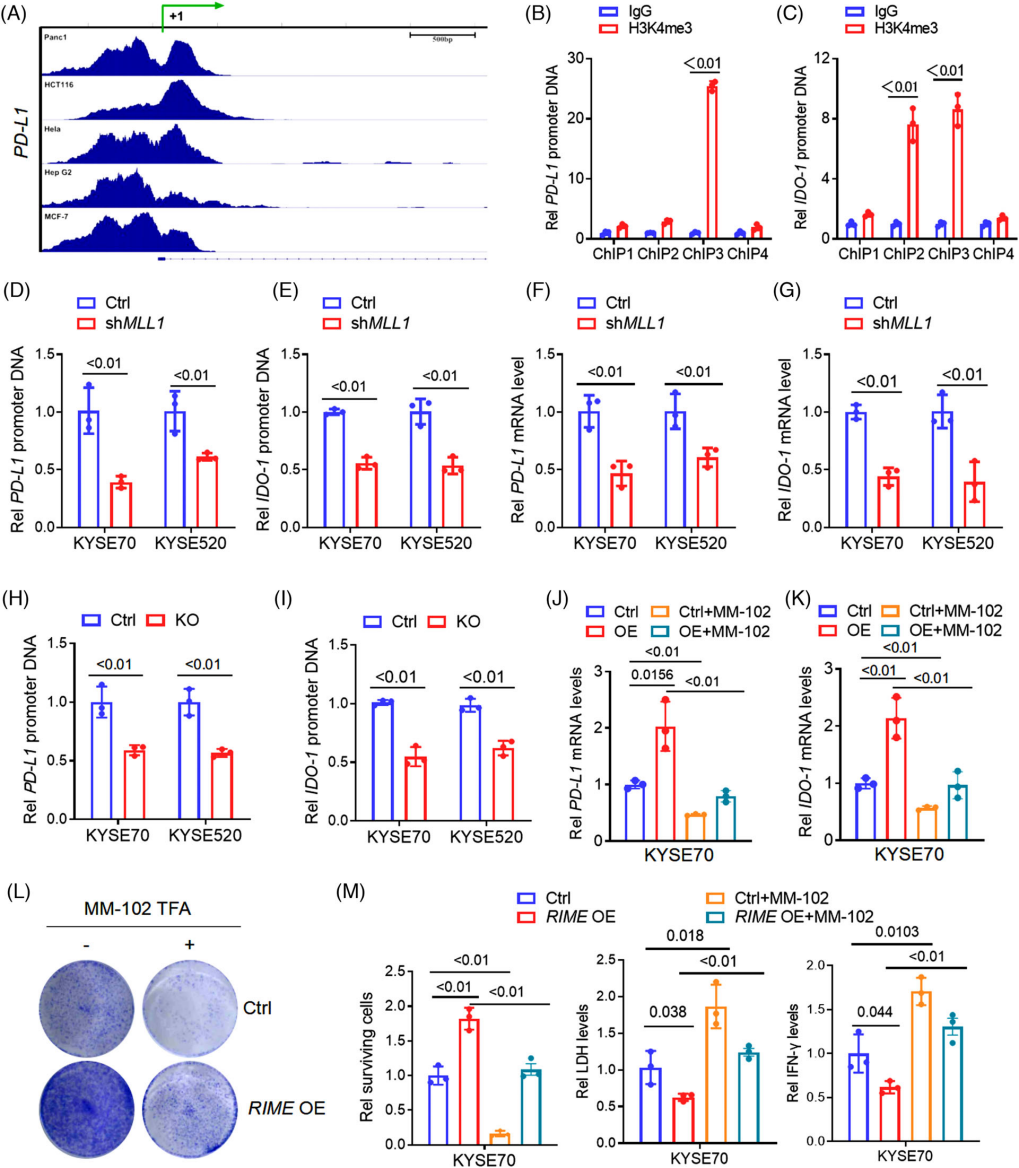
*RIME* regulates MLL1‐H3K4me3‐mediated PD‐L1/IDO‐1 expression. (A) ChIP‐seq analysis showed that H3K4me3 levels were enriched in the *PD‐L1* promoter regions. The ChIP‐seq data was obtained from ENCODE database. (B‐C) ChIP assays indicated that H3K4me3 marks were enriched in the *PD‐L1* promoter region around −1000 to 0 bp (ChIP1 to ChIP4 are primers designed for each fragment, representing −3000 to −2000 bp, −2000 to −1000 bp, −1000 to 0 bp, and 0 to +1000 bp, respectively), and in the IDO‐1 promoter region around −1500 to −500 bp (ChIP1 to ChIP4 represented −2000 to 0 bp). (D‐E) ChIP and quantitative real‐time polymerase chain reaction (qRT‒PCR) analysis revealed that MLL1 depletion by shRNA dramatically decreased H3K4me3 levels in the *PD‐L1* and *IDO‐1* promoter regions. (F, G) qRT‒PCR analysis revealed that MLL1 depletion by shRNA dramatically decreased *PD‐L1* and *IDO‐1* mRNA levels in oesophageal squamous cell carcinoma (ESCC) cells. (H, I) ChIP and qRT‒PCR analysis revealed that *RIME* KO significantly decreased the H3K4me3 level in the *PD‐L1* and *IDO‐1* promoter regions. (J, K) qRT‒PCR analysis showed that the increased *PD‐L1* and *IDO‐1* expression levels induced by *RIME* overexpression were abolished by MLL1 inhibition (MM‐102 treatment). (L‐M) Crystal violet staining assay showed that MLL1 inhibition abolished the resistance of ESCC cells to cytotoxic CD8^+^ T cells induced by *RIME* overexpression. The decreased LDH and IFN‐γ levels induced by *RIME* overexpression in the ESCC‐T cell co‐culture medium were also abolished by MLL1 inhibition.

Studies have reported that MLL1 mediates H3K4 trimethylation (me3) in the *PD‐L1* promoter region and promotes PD‐L1 expression.^25^ To explore the epigenetic mechanisms that regulate PD‐L1 and IDO‐1 expression in ESCC cells, we performed bioinformatics analysis using Chromatin immunoprecipitation (ChIP) sequencing data from the ENCODE database and found that in various malignancies, such as pancreatic cancer, colorectal cancer, cervical cancer, hepatic cell carcinoma, and breast cancer, H3K4me3 marks were enriched in both the *PD‐L1* and *IDO‐1* promoter regions around the transcription start site (Figure 4A and Figure S4A). ChIP‐qPCR assays indicated that H3K4me3 levels were enriched in the *PD‐L1* promoter region at approximately −1000 to 0 bp and in the *IDO‐1* promoter region at approximately −1500 to −500 bp in ESCC cells (Figure 4B,C and Figure S4B,C). ChIP and qPCR analysis revealed that *MLL1* depletion by shRNA dramatically decreased H3K4me3 levels in the *PD‐L1* and *IDO‐1* promoter regions, as well as *PD‐L1* and *IDO‐1* mRNA levels in human ESCC cells (Figure 4D–G and Figure S4D). Consistent with our previous results that *RIME* KO notably downregulated *PD‐L1* and *IDO‐1* expression, *RIME* KO significantly decreased the H3K4me3 level in the *PD‐L1* and *IDO‐1* promoter regions (Figure 4H,I). Therefore, we speculated that *RIME* regulates MLL1‐H3K4me3‐mediated *PD‐L1* and *IDO‐1* expression to evade immune surveillance in ESCC cells.

MM‐102 is a specific MLL1 inhibitor used to block the interaction between WDR5 and MLL1.^28^ To prove our above hypothesis, we treated ESCC cells with MM‐102 and observed that the increased *PD‐L1* and *IDO‐1* expression levels induced by *RIME* overexpression were abolished by MLL1 inhibition (Figure 4J,K and Figure S4E,F). A crystal violet staining assay showed that MLL1 inhibition also abolished the resistance of ESCC cells to cytotoxic CD8^+^ T cells induced by *RIME* overexpression (Figure 4L,M). Consistently, the decreased LDH and IFN‐γ levels induced by *RIME* overexpression in the ESCC‐T coculture medium were also abolished by MLL1 inhibition (Figure 4M and Figure S4G). Overall, *RIME* regulates MLL1‐H3K4me3‐mediated PD‐L1 and IDO‐1 expression, resulting in more cellular resistance to the cytotoxicity of CD8^+^ T cells and thus facilitating ESCC immune evasion.

### 2.7 *RIME* inhibition enhanced antitumour immunity in ESCC treatment

**FIGURE 5:**
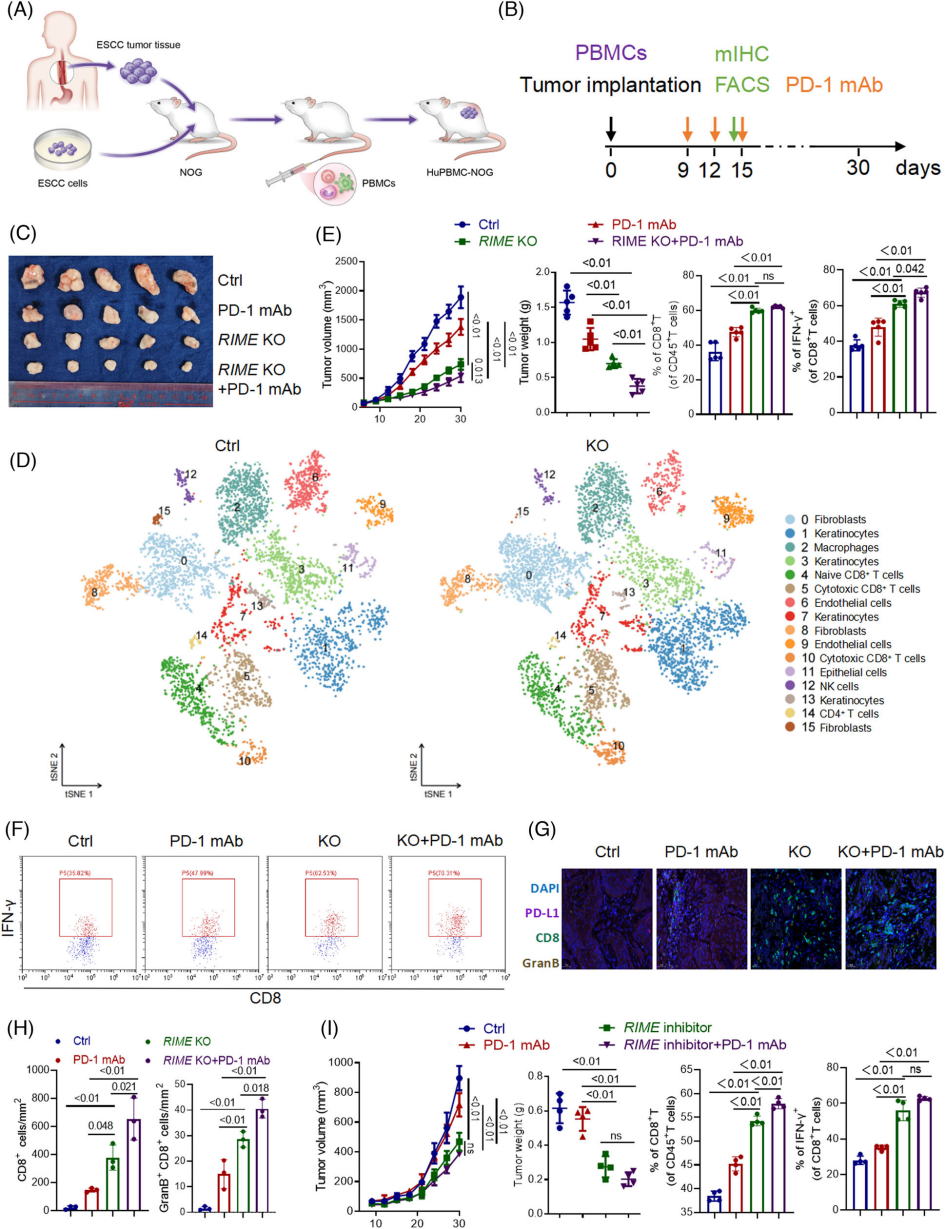
*RIME* inhibition enhanced antitumour immunity in oesophageal squamous cell carcinoma (ESCC) treatment. (A, B) Graphic illustration of the construction of Hu‐PBMC‐NOG and time points of administration and sampling. (C) Xenografts from the Hu‐PBMC‐NOG mouse model were collected and photographed on day 30. *RIME* CRISPR KO dramatically repressed tumour growth and improved the anti‐tumour efficacy of PD‐1mAb. (D) ScRNA‐seq data of xenografts from the Hu‐PBMC‐NOG mouse model was aligned and quantified using the CellRanger toolkit v.3.1. Major cell types in the tumour tissue were clustered using Seurat (v.3.2.3). ScRNA‐seq analysis of the xenografts suggested that *RIME* KO increased CD8^+^ T cells and cytotoxic CD8^+^ T cells. (E) Flow cytometry analysis showed that *RIME* KO increased the proportion of CD8^+^ T cells and IFN‐γ^+^ CD8^+^ T cells in the ESCC xenografts. (F) Representative flow cytometry results showed that *RIME* KO increased the proportion of IFN‐γ^+^ CD8^+^ T cells in the ESCC xenografts. (G‐H) Multiplex fluorescent immunohistochemistry assays showed that *RIME* KO increased the proportion of CD8^+^ T cells and GranB^+^ CD8^+^ T cells in the ESCC xenografts. Scale bar, 50 μm. (I) As indicated, Hu‐PBMC‐NOG‐PDX mice were injected with Ctrl or *RIME* inhibitor (10 nmol per injection) with or without PD‐1 mAb (200 μg per injection). It showed that targeting *RIME* significantly inhibited tumour development. The *RIME* inhibitor increased the proportion of CD8^+^ T cells and IFN‐γ^+^ CD8^+^ T cells in the ESCC patient‐derived xenografts.

According to the Ensembl database,^29^ lncRNA *RIME* does not have a homologous lncRNA in mice; the functional role of *RIME* could not be studied in immunogenic mice. Therefore, ESCC tumour tissues or ESCC cells were transplanted subcutaneously into NOG mice to establish patient‐derived xenograft (PDX) or cell line‐derived xenograft (CDX) models. Human peripheral mononuclear cells (hu‐PBMCs), which were pre‐primed for a humanized mouse model, were intravenously administered into NOG mice to better simulate a human immunological milieu in vivo.^19^, ^22^ Flow cytometry analysis showed that the percentage of human CD45^+^ cells in the mouse blood exceeded 60%, indicating the successful construction of the huPBMC‐NOG‐CDX/PDX model (Figure 5A,B and Figure S5A). qRT‒PCR analysis of xenografts verified that *RIME* KO efficiently suppressed *RIME* expression in vivo (Figure S5B). In the huPBMC‐NOG‐CDX model, *RIME* CRISPR KO significantly reduced tumour development and showed a better antitumour effect than PD‐1 mAb monotherapy (Figure 5C). The reason why *RIME* KO has better outcomes than PD‐1 mAb may be related to the fact that *RIME* regulates the expression of multiple immunosuppression‐related molecules, not just PD‐L1. To further observe how *RIME* affects immune cell subpopulations within the tumour microenvironment, xenografts were collected and single‐cell RNA sequencing (scRNA‐seq) analysis was carried out. Consistent with our in vitro experiments, scRNA‐seq analysis of the xenografts suggested that *RIME* KO increased CD8^+^ T cells and cytotoxic CD8^+^ T cells (Figure 5D and Figure S5C,D). Flow cytometry and multiplex fluorescent immunohistochemistry (mIHC) further verified that *RIME* KO increased CD8^+^ T cells, IFN‐γ^+^ and granzyme B (GranB)^+^ CD8^+^ T cells in ESCC xenografts (Figure 5E–H and Figure S5E).

To further explore the potential effects of *RIME* on other immune cells infiltrating the tumour microenvironment, we performed flow cytometry analysis of CD4^+^ T cells, M1 macrophages, M2 macrophages, and NK cells in the xenografts (Figure S5F–I). Surprisingly, we demonstrated that *RIME* KO decreased the proportion of pro‐tumour M2 macrophages in ESCC xenografts while increasing CD4^+^ T cells and anti‐tumour M1 macrophages proportion in ESCC xenografts. Moreover, *RIME* KO had no effect on the proportion of NK cells in the xenografts. These data implied that *RIME* is a critical regulator of the tumour immune microenvironment, *RIME* KO favours anti‐tumour immune responses by remodelling multiple tumour‐infiltrating immune cells.

We also designed a *RIME* inhibitor with a strong depletion effect on *RIME*, which could be used in vivo and in vitro (Figure S5J,K). In our huPBMC‐NOG‐PDX model, targeting *RIME* significantly inhibited tumour development and showed a better antitumour effect than PD‐1 mAb monotherapy (Figure 5I and Figure S5L). The *RIME* inhibitor also increased the proportion of CD8^+^ T cells and cytotoxic CD8^+^ T cells in ESCC patient‐derived xenografts (Figure 5I), suggesting that *RIME* is a promising therapeutic target.

### 2.8 The *RIME*‐MLL1‐H3K4me3 axis is clinically associated with ESCC development

**FIGURE 6:**
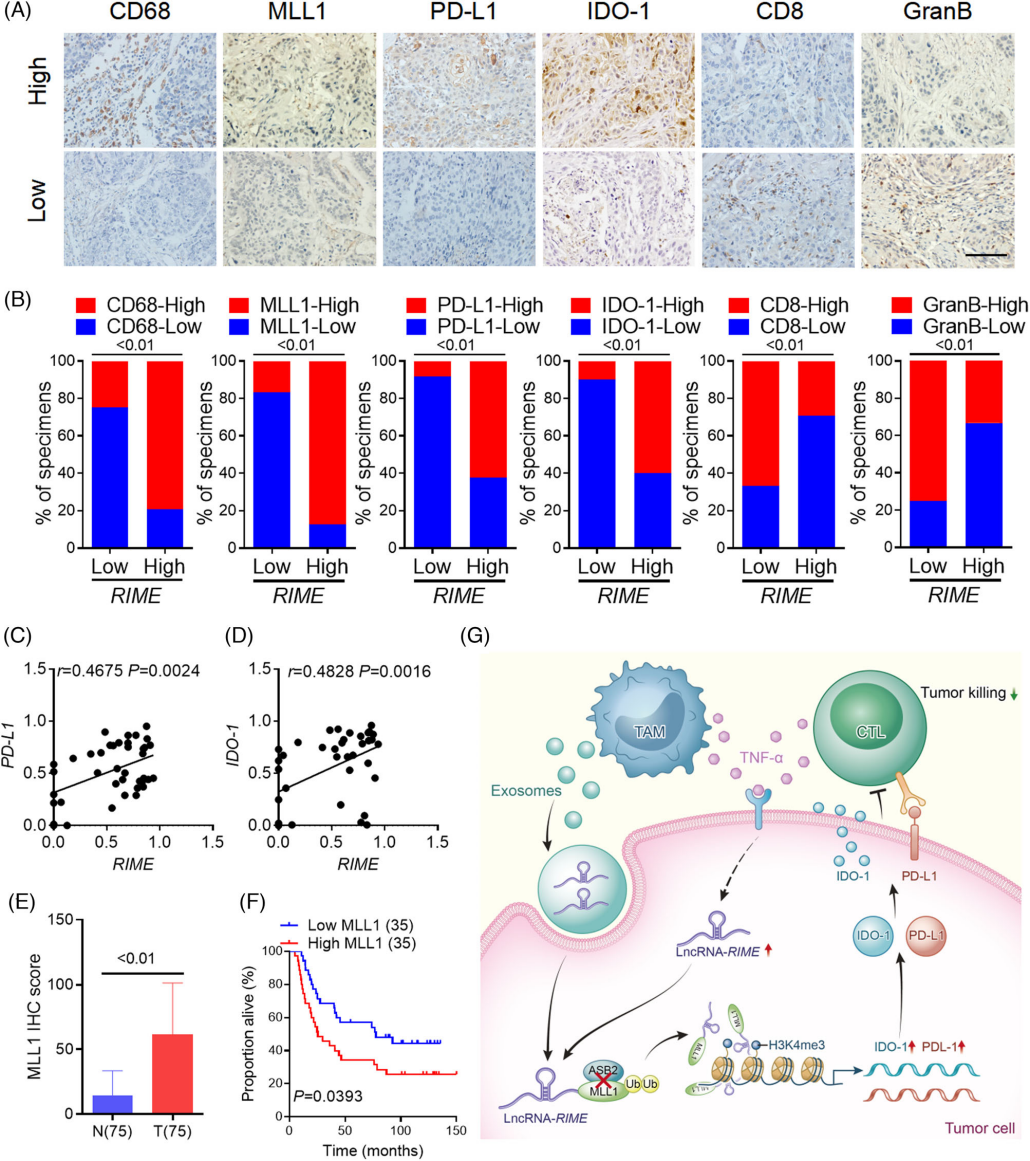
The *RIME*‐MLL1‐H3K4me3 axis is clinically associated with oesophageal squamous cell carcinoma (ESCC) development. (A) Representative images of IHC assays in ESCC tissues with low or high *RIME* expression. The *RIME*‐high group exhibited higher CD68, MLL1, PD‐L1 and IDO‐1 expression but lower CD8 and GranB expression. Scale bar, 100 μm. (B) Percentage of tumour samples with the indicated markers in groups with low or high *RIME* expression. The *RIME*‐high group exhibited higher CD68, MLL1, PD‐L1, and IDO‐1 expression but lower CD8 and GranB expression. (C, D) Quantitative real‐time polymerase chain reaction (qRT‒PCR) and Pearson correlation analysis showed that *RIME* and *PD‐L1/IDO‐1* expression in ESCC tissues were closely related. (E) IHC score analysis showed that MLL1 expression was markedly upregulated in malignant tissues than in para‐carcinoma tissues. (F) Overall survival analysis showed that high MLL1 expression correlated with poor outcomes in ESCC patients (log‐rank test). (G) Schematic showing the mechanism by which long noncoding RNA (lncRNA) *RIME* regulates MLL1‐H3K4me3‐mediated immune evasion.

To verify that the *RIME*‐MLL1‐H3K4me3 axis is clinically associated with ESCC development, we performed immunohistochemistry analysis and evaluated CD68, MLL1, PD‐L1, IDO‐1, CD8 and GranB expression in tumour tissues from a cohort of ESCC patients (SYSUCC, *n* = 70). The group with elevated *RIME* expression demonstrated increased expression of CD68, MLL1, PD‐L1 and IDO‐1 but reduced expression of CD8 and GranB, which was the opposite in the *RIME* low expression group (Figure 6A,B). qPCR analysis also confirmed that *RIME* and *PD‐L1*/*IDO‐1* expression in ESCC tissues were closely related (Figure 6C,D). Furthermore, IHC staining was applied to assess the expression of MLL1 in ESCC and para‐carcinoma tissues. MLL1 expression was markedly upregulated in malignant tissues, and high MLL1 expression correlated with poorer prognosis in ESCC patients (Figure 6E,F). These data further confirmed that the *RIME*‐MLL1‐H3K4me3 axis plays a pivotal role in disrupting antitumour immunity in ESCC patients (Figure 6G).

## 3 DISCUSSION

Immune checkpoint inhibitors (ICIs) have considerably enhanced the prognosis of ESCC patients in recent years, but to date, effective predictive biomarkers for immunotherapy response remain controversial and challenging.^5^, ^6^, ^30^ Studies have reported that exosome‐transmitted lncRNAs promote malignant tumour function and are associated with poor chemotherapeutic response and shorter survival of cancer patients.^31^ A prospective clinical trial showed that two small RNAs in salivary exosomes, tRNA‐GlyGCC‐5 and sRESE, have shown promise as noninvasive biomarkers for the diagnosis and prognosis of ESCC and could be used as preoperative biomarkers to screen individuals who might benefit from adjuvant therapy.^32^ Our research further highlights the crucial role of exosomal lncRNAs in intercellular communication, especially between tumour cells and TAMs. High expression of lncRNA *RIME* was linked to poor outcomes in ESCC patients, suggesting that *RIME* may be an appealing immunotherapy prediction biomarker. The use of *RIME* as a biomarker for immunotherapy could prevent unnecessary treatment costs and drug toxicity in nonresponders.

Recent studies have shown that in a variety of cancers, multiple epigenetic modifiers, such as SET domain bifurcated histone lysine methyltransferase 1 (SETDB1) and lysine‐specific demethylase 1A (LSD1), play critical roles in regulating the expression of endogenous antigens and immune responses.^33^ H3K4 demethylase LSD1 deletion activates the interferon signalling pathway by stimulating endogenous retrovirus (ERV) elements, which enhances the antitumour effect of T cells. A previous study revealed that H3K4me3 is enriched in the *PD‐L1* promoter in pancreatic tumour cells, and MLL1 directly binds to the *PD‐L1* promoter and catalyses H3K4me3 to activate *PD‐L1* transcription in tumour cells. MLL1 inhibition in combination with immunotherapy effectively suppressed pancreatic tumour growth. In many preclinical models, DNA methyltransferase inhibitors (DNMTs) in combination with ICIs greatly improved antitumour efficacy by reversing T‐cell exhaustion. However, broad‐spectrum inhibition of DNA methylation also impairs the function of normal cells. Therefore, more precise and effective strategies are needed to rescue epigenetic modifier‐mediated immunosuppression.

Our study demonstrated that *RIME* binds to MLL1 and prevents ASB2‐mediated ubiquitination, which enhances MLL1 stability and increases the enrichment of H3K4me3 in the *PD‐L1* and *IDO‐1* promoter regions, thus upregulating tumoral expression of immune checkpoint markers PD‐L1/IDO‐1. This upregulation contributes to T‐cell dysfunction and blocks the antitumour immune response, which facilitates immune evasion and tumour development. Since *RIME* is induced by TNF‐α and upregulated in cancer cells, targeting *RIME* might reduce the adverse effect of epigenetic modifier inhibitors on normal cells and has tremendous potential to improve antitumour immune responses. However, it also needs to be considered that targeting *RIME* and MLL1 is associated with risks of damaging normal cells. Altering DNA methylation of important genes may disrupt a wide range of physiological processes, notably DNA repair, cellular growth, differentiation, and programmed cell death, resulting in hematotoxicity, nephrotoxicity, hepatotoxicity, gastrointestinal toxicity, and cardiovascular toxicity, amongst others.^34^, ^35^ These epigenetic changes may also impede immune cell development, differentiation, activation, and cytokine production, thereby affecting clinical response to immunotherapy.^33^ This study preliminarily explored the effects of *RIME* on several tumour‐infiltrating immune cells, and more research is required to thoroughly reveal the functionality of *RIME* in somatic cells.

Oligonucleotide‐based therapy has recently garnered the interest of the pharmaceutical industry as a promising avenue for the development of new medication. Antisense oligonucleotides (ASOs), short interfering RNAs (siRNAs), microRNAs (miRNAs), and aptamers, are examples of oligonucleotide therapies that can be used to treat a range of genetic illnesses, including neurodegenerative disorders, respiratory disorders, and cancer.^36^, ^37^ By the end of 2022, fifteen drugs that were developed using oligonucleotides have already been approved by the US Food and Drug Administration. Moreover, there are over 246 clinical trials registered, among which 22 are phase 3 trials. In this study, we found that inhibiting *RIME* with CRISPR technology or antisense oligonucleotides significantly repressed tumour development in huPBMC‐NOG mice, which suggests that developing drugs that target *RIME* carries significant translational value and offers new approaches to enhance anti‐tumour therapy.

To improve the specificity of the *RIME* CRISPR KO strategy and minimize potential off‐target effects, we used a dual sgRNA/Cas9 all‐in‐one system to generate *RIME* KO cells. Sanger sequencing and qPCR analysis were used to validate the “on‐target” effect of *RIME* CRISPR/Cas9 sgRNA. ASOs used in this study also showed a strong depletion effect on *RIME* both in vivo and in vitro. However, CRISPR technology and ASOs still have potential off‐target effects; the sgRNA or oligonucleotide sequence may be partially similar to unintended target sequences and disturb other gene expression, leading to unexpected side effects. To date, there has been no well‐validated method for accurately predicting off‐target mutations,^38^ and further studies should consider the off‐target related toxicities of *RIME*‐targeting drugs. Besides, numerous obstacles exist in the development of *RIME*‐targeting drugs. The first is the instability. Naked oligonucleotides are prone to be degraded by RNases and removed from the circulation before they can play a role. Appropriate chemical modification is a reliable method for enhancing the delivery efficiency of oligonucleotide drugs. The delivery systems of *RIME*‐targeting drugs remain challenging. Many drug vehicles have been investigated; the poor plasma clearance makes nanoparticles difficult to deliver to target tissues.^39^ Other delivery systems such as lipid‐based delivery systems, show high delivery efficiency but are often associated with systemic toxicity. Notably, exosomes are promising lipid nanocarriers with a high degree of biocompatibility and the potential to selectively target endogenous cellular ligands.^40^

The mechanisms of immunotherapy resistance that have been described include loss of expression of tumour antigen expression, lack of antigen presentation, constitutive PD‐L1 expression, and T‐cell dysfunction. Here, we demonstrated that TAM exosome transmission and TNF‐α stimulation in the tumour microenvironment induce *RIME* upregulation in tumour cells, which constitutively upregulates the expression of the immune checkpoint markers PD‐L1/IDO‐1 and results in antitumour T‐cell inactivation and a reduced response to anti‐PD‐1 mAb therapy. This study reveals lncRNA‐mediated tumour immunosuppression and immunotherapy resistance and provides important insights for the improvement of immunotherapy responses in ESCC patients.

## 4 MATERIALS AND METHODS

### 4.1 Cell lines

Human oesophageal cancer cell lines (KYSE30, KYSE150, KYSE70 and KYSE520) were purchased from the German Cell Culture Collection (DSMZ). The cells were cultured in RPMI‐1640 medium that was supplemented with 10% FBS and 1% penicillin/streptomycin, and the temperature was kept at 37°C with 5% CO2. The cells underwent authentication by short tandem repeat (STR)‐PCR DNA profiling where the results showed that none of the cells were contaminated with mycoplasma. TAMs were isolated from fresh ESCC patient samples as previously described.^7^ TAMs (∼1×10^6^ cells/well) were plated on 0.4‐μm Transwell inserts. ESCC cells (∼2×10^5^ cells/well) were seeded on 6‐well plates. TAMs and ESCC cells were cocultured for 72 h before analysis.^13^

### 4.2 Plasma and tissue specimens

All plasma and tissue specimens were obtained from SYSUCC (Guangzhou, China). All patients gave written informed consent before having their samples taken. The study complied with the Declaration of Helsinki and received approval from the Institutional Review Board of SYSUCC. The clinicopathological information is provided in Table S4.

### 4.3 RNA extraction, qRT–PCR, and RNA sequencing

The differential centrifugation method was used to isolate exosomes from plasma.^41^ RNA sequencing and bioinformatics were performed to analyse exosomal lncRNAs (RiboBio). The isolation of total RNA was carried out utilizing the TRIzol reagent. The resulting complementary DNA was analysed by qRT‐PCR performed with the qPCR Master Mix Kit (Promega). The 2*^−ΔCt^* or 2*^−ΔΔCt^* method was used to analyze relative gene expression. The details of the primer sequences utilized are in Table S5.

### 4.4 CRISPR/Cas9

*RIME* KO cells were constructed as previously reported.^13^, ^20^ The expression vectors provided by Kidan Biotechnology Co., Ltd. were transfected into ESCC cells. Stable transfectants were selected using puromycin for one week. The *RIME* knockout efficiency of isolated colonies was analysed by PCR analysis. Table S6 contains a list of the sgRNA sequences.

### 4.5 Plasmid and cell transduction

Plasmids expressing MLL1 truncated mutants with FLAG‐tags and lentivirus for *RIME* overexpression were provided by OBiO Technology. Sequencing confirmed the integrity of the constructs. Plasmids were transfected into cells using Lipofectamine 3000 Reagent (Thermo Scientific) in accordance with the manual provided. Lentivirus was transduced into cells in the presence of polybrene (MedChemExpress).

### 4.6 T‐cell cytotoxicity

Purified human CD8^+^ T cells were cocultured with ESCC cells at an E/T ratio of 10:1. After 12 hours, the supernatant was collected, and the IFN‐γ and IL‐2 levels were determined using ELISA kits (ab174443, ab270883, Abcam). Lactate Dehydrogenase(LDH) Assay Kit (Cytotoxicity) (ab102526, Abcam) was used to measure LDH activity. Tumour cells were washed and subjected to a crystal violet staining assay (Beyotime).

### 4.7 RNA pull‐down, RIP and MTRAP assays

RNA pull‐down, RIP, and MTRAP assays were carried out according to previous reports.^13^, ^21^, ^26^ Briefly, *RIME* was transcribed and biotinylated in vitro using a MEGAscript T7 Transcription Kit and a Pierce RNA 3′ End Desthiobiotinylation Kit. Pierce Magnetic RNA‒Protein Pull‐Down Kit was used for RNA pull‐down assays. The Magna RBP Immunoprecipitation Kit was utilized to conduct RIP assays in accordance with the manufacturer's guidelines. 400 mJ/cm^2^ of 254 nm UV light was used to irradiate cells cotransfected with pcDNA3.1‐MS2/pcDNA3.1‐MS2‐*RIME* and MCP‐3xFLAG plasmids in the MTRAP assay. Subsequently, the IP assays were performed with anti‐FLAG antibody. The MS2‐*RIME* binding protein complex was detected by IB.

### 4.8 FISH, IF, and Duolink in situ proximity ligation assay

FISH assays were carried out using a Fluorescent in Situ Hybridization Kit (RiboBio). IF staining was performed as described in our previous study.^13^, ^20^ Duolink in situ proximity ligation assay (PLA) (Sigma‒Aldrich DUO92101) was performed according to the manufacturer's instructions and was used to examine molecular proximity. A Zeiss LSM 880 microscope and Zeiss Zen 2.3 lite software were used in our confocal microscopy experiments (Carl Zeiss).

### 4.9 Humanized PBMC mouse model

The humanized PBMC (hu‐PBMC) mouse model was established as previously described.^22^ Beijing Vital River Laboratories (Beijing, China) supplied the NOG mice. Shanghai AoNeng Biotechnology (Shanghai, China) provided the peripheral blood mononuclear cells (PBMCs) primed for the humanized mouse model used in our in vivo experiments. Six‐week‐old female mice were transplanted with 5×10^6^ PBMCs by tail intravenous injection. Quantification of the percentages of human CD45^+^ cells in the peripheral blood was used to determine the levels of engraftment.^42^ CDX and PDX models were established as previously described.^13^, ^21^ The mice were subcutaneously injected with 2×10^6^ ESCC cells or ESCC patient‐derived xenografts in the flank. Measurements of the tumour were carried out every three days. Nine days post‐inoculation, PD‐1 mAb or IgG (200 μg per injection; Shanghai Junshi Biosciences Co., Ltd) was intraperitoneally injected into the mice every 3 days. For the hu‐PBMC‐PDX model, intravenous injection of 10 nmol *RIME* inhibitor or control (RiboBio) was carried out at 3‐day intervals. After two injections, the tumours were collected for mIHC, flow cytometry, or single‐cell sequencing.

### 4.10 Flow cytometry analysis

Fresh tumour samples were collected as previously described.^19^ Samples were chopped into smaller pieces and then digested with a Tumour Dissociation Kit (Miltenyi Biotec) and incubated at 37°C for 30 min. After being washed with a FACS buffer and gentle centrifugation, the cell pellets were stained with Zombie Aqua, anti‐human CD45, TCR and CD8 antibodies for 20 min at 4°C. After fixation and permeabilization by BD Cytofix/Cytoperm Fixation and Permeabilization Solution, the cells were stained with anti‐human IFN‐γ antibodies for 30 min at 4°C, washed, and detected by a cytoFLEX flow cytometer, and the data were analysed using CytExpert software (Beckman Coulter). Table S2 lists the flow cytometry analysis antibodies.

### 4.11 scRNA‐seq analysis

Single‐cell suspensions were obtained as previously described.^43^ Briefly, fresh tumour samples were cut into pieces and digested with a MACS tumour dissociation kit (Miltenyi Biotec). Single‐cell suspensions were collected, and a Rigel S3 fluorescence cell analyser (Countstar) was used to assess cell viability and concentration. The cell suspension was loaded onto the Chromium single‐cell controller (10x Genomics). The scRNA‐seq libraries were constructed using the Single Cell 3′ Library and Gel Bead Kit v3.1 and sequenced using the Illumina NovaSeq 6000 sequencer (performed by CapitalBio Technology). scRNA‐seq data were aligned and quantified using the CellRanger toolkit v.3.1. Major cell types in the tumour tissue were clustered using Seurat (v.3.2.3). Differentially expressed genes were identified using FindMarkers. Enrichment analysis was performed using KOBAS software with Benjamini‒Hochberg multiple testing adjustment using the top 20 marker genes of the clusters. The outcomes were graphically represented utilizing the R software package.

### 4.12 mIHC and IHC

mIHC and IHC were carried out in accordance with prior reports.^44^, ^45^ In the IHC assays, scores of markers were determined by the intensity and proportion of positive cells. mIHC was performed with a TSA 7‐colour kit (abs50015‐100T; Absinbio). The slides were deparaffinized, and incubated with the indicated antibodies for 30 min and before being exposed to a secondary antibody (Absinbio) for 10 min. Slides were labelled with fluorescent dyes for 10 min, washed with TBST buffer, moved into a preheated citrate solution (90°C) and finally heated in a microwave for 15 min. Then the slides were cooled, stained with DAPI (abs47047616, Absinbio, Shanghai), and rinsed in distilled water. Pannoramic MIDI II (3DHISTECH) was used to take pictures, and Indica Halo software was used to analyse images.

### 4.13 Statistics

All experiments were carried out three times, and representative data and images are shown. The results are presented as the mean ± SD. Student's t test was used to compare significant differences between two groups. One‐way ANOVA was used to determine the significant differences among three or more groups. Pearson's correlation analysis was used to assess the correlation between two indicators. The log‐rank test was used to evaluate the OS and PFS data. Fisher's exact test was used to compare proportions among different groups. Statistical analysis was performed using GraphPad version 8.0. *p* < 0.05 was considered statistically significant.

## CONFLICT OF INTEREST STATEMENT

The authors declare no conflict of interest.

## Supporting information

Supporting Information

Supporting Information

## Data Availability

RNA‐seq data for the ESCC cell lines are available in Sequence Read Archive (SRA) PRJNA732981 (http://bigd.big.ac.cn/gsa‐human/). The ChIP‐seq data was obtained from the ENCODE database (https://www.encodeproject.org/). The information on key resources is listed in Table S7. Additional data that corroborates the results of our study can be found in the Supplementary Information Files. Alternatively, interested parties may request additional information directly from the corresponding authors, subject to reasonable requests.
