# Supplementary material for: Long noncoding RNA Regulating ImMune Escape regulates mixed lineage leukaemia protein‐1‐H3K4me3‐mediated immune escape in oesophageal squamous cell carcinoma
Source: Clin Transl Med. 2023 Sep 15;13(9):e1410. doi: 10.1002/ctm2.1410 (PMC10502462; doi:10.1002/ctm2.1410)
Supplement: Supplementary file 1 — Supporting Information [file CTM2-13-e1410-s002.pdf]

Figure S1

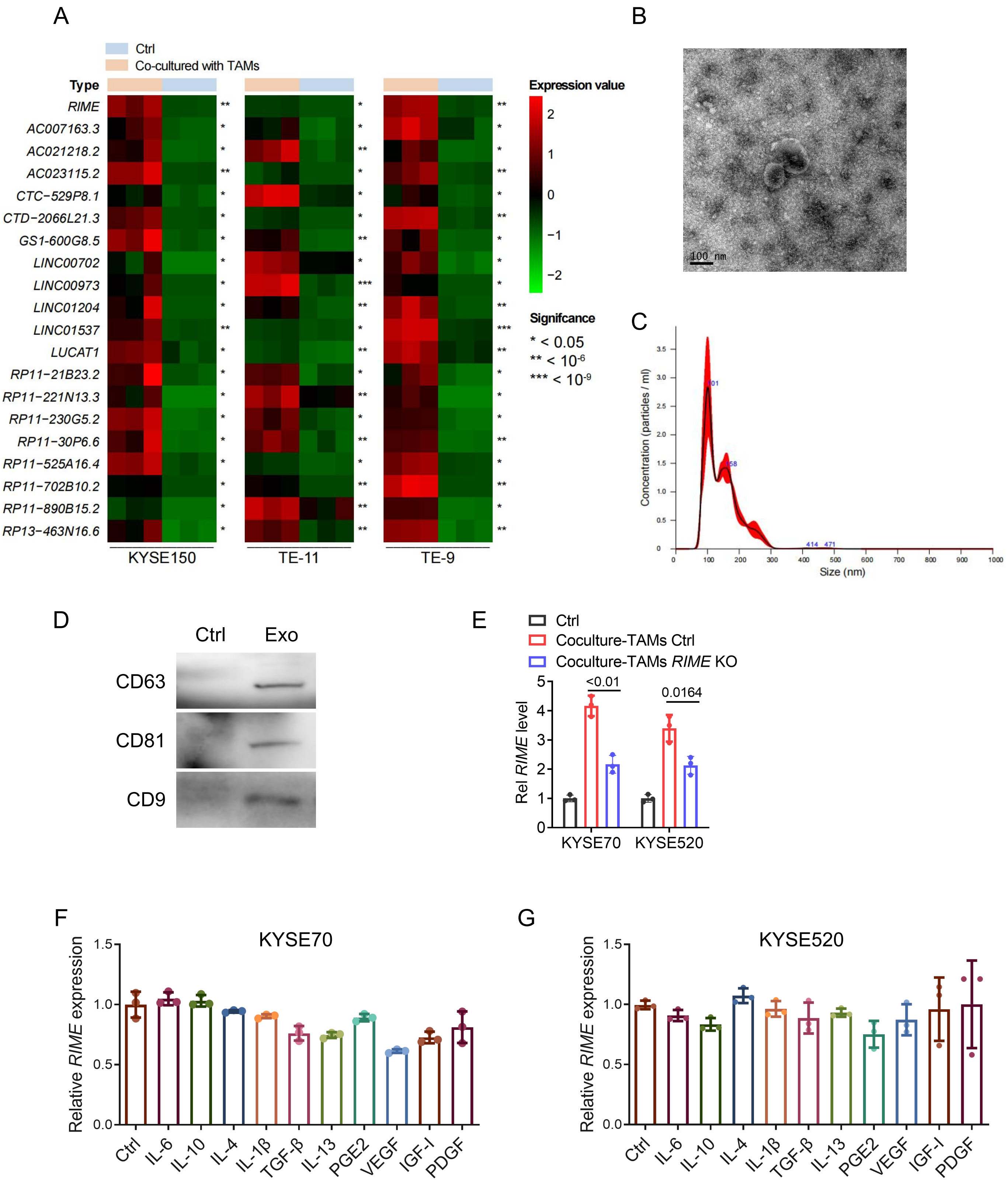

A
Ctrl
Co-cultured with TAMs
Type
RIME
AC007163.3
AC021218.2
AC023115.2
CTC−529P8.1
CTD−2066L21.3
GS1-600G8.5
LINC00702
LINC00973
LINC01204
LINC01537
LUCAT1
RP11−21B23.2
RP11−221N13.3
RP11−230G5.2
RP11−30P6.6
RP11−525A16.4
RP11−702B10.2
RP11−890B15.2
RP13−463N16.6
KYSE150
TE-11
TE-9
Expression value
2
1
0
−1
−2
Signifcance
* < 0.05
** < 10-6
*** < 10-9
B
100 nm
C
Concentration (particles / ml)
Size (nm)
D
Ctrl
Exo
CD63
CD81
CD9
E
Ctrl
Coculture-TAMs Ctrl
Coculture-TAMs RIME KO
Rel RIME level
<0.01
0.0164
KYSE70
KYSE520
F
KYSE70
Relative RIME expression
Ctrl
IL-6
IL-10
IL-4
IL-1β
TGF-β
IL-13
PGE2
VEGF
IGF-I
PDGF
G
KYSE520
Relative RIME expression

Figure S1 LncRNA *RIME* correlates with immunotherapy outcomes

(**A**) TAM-associated lncRNAs were identified by performing RNA-seq analysis of ESCC cells cocultured with TAMs. **(B)** Electron microscope observation of exosomes isolated from plasma. Scale bar, 100 nm. **(C)** Nanoparticle tracking analysis of exosomes isolated from plasma. **(D)** Immunoblotting analysis of CD63, CD81, and CD9 expression in control (Ctrl, no exosome fraction) and plasma exosome fraction (Exo)**.** Panel **B**-**D** demonstrated that exosomes were identified as extracellular vesicles in the approximate size of 100 nm in diameter, and contained typical exosomal markers such as CD63, CD81, and CD9. **(E)**The qRT–PCR analysis showed that *RIME* expression was upregulated in ESCC cells cocultured with TAMs, while this effect was attenuated when cocultured with *RIME* KO TAMs. **(F-G)** qRT–PCR analysis of *RIME* expression in KYSE70 and KYSE520 cells treated with various cytokines routinely secreted by TAMs, which showed that TNF-α stimulation upregulated *RIME* expression in ESCC cells.

Figure S2

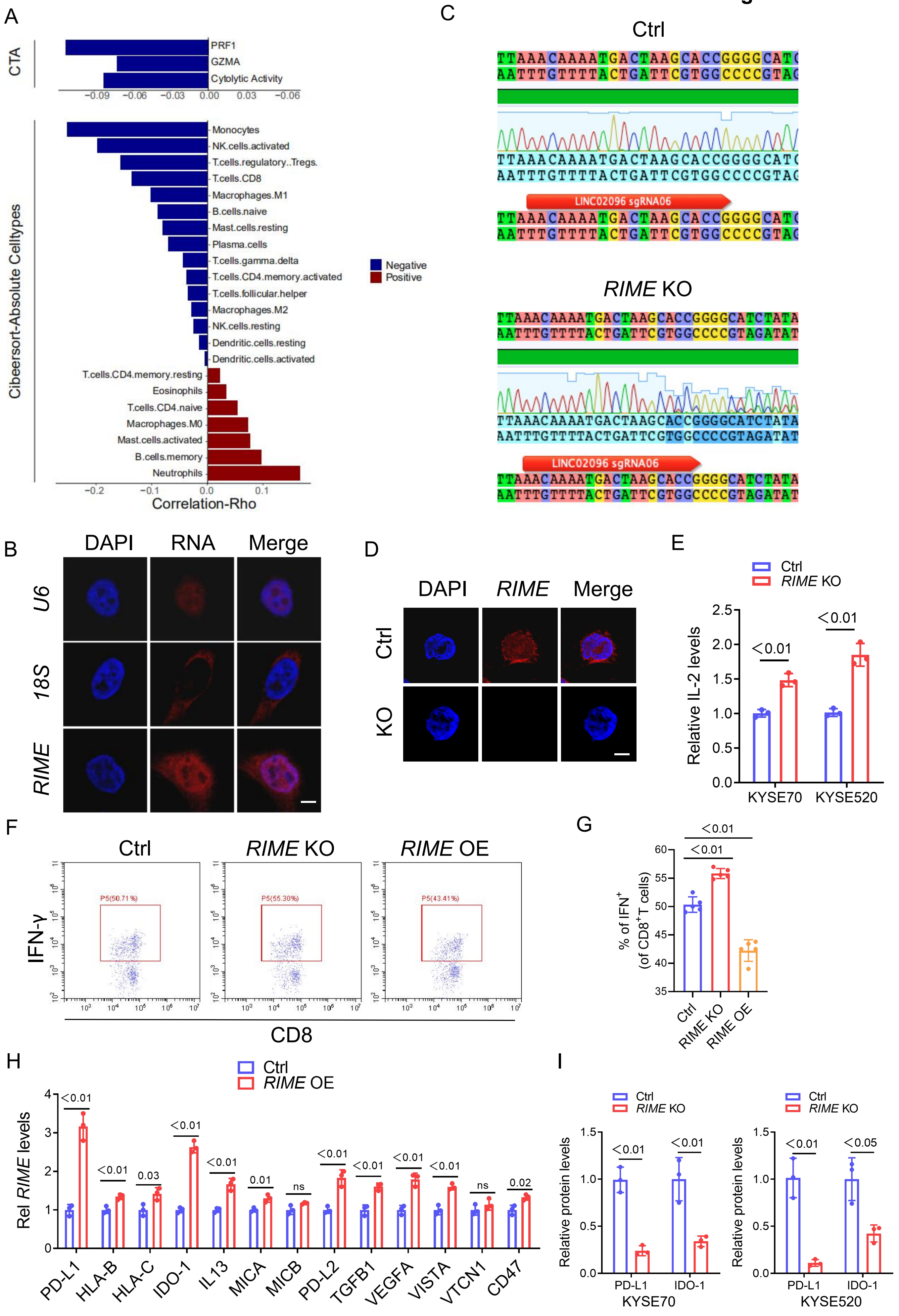
A
CTA
PRF1
GZMA
Cytolytic Activity
Cibeersort-Absolute Celltypes
Monocytes
NK.cells.activated
T.cells.regulatory..Tregs.
T.cells.CD8
Macrophages.M1
B.cells.naive
Mast.cells.resting
Plasma.cells
T.cells.gamma.delta
T.cells.CD4.memory.activated
T.cells.follicular.helper
Macrophages.M2
NK.cells.resting
Dendritic.cells.resting
Dendritic.cells.activated
T.cells.CD4.memory.resting
Eosinophils
T.cells.CD4.naive
Macrophages.M0
Mast.cells.activated
B.cells.memory
Neutrophils
Correlation-Rho
Negative
Positive
B
DAPI
RNA
Merge
U6
18S
RIME
C
Ctrl
LINC02096 sgRNA06
RIME KO
LINC02096 sgRNA06
D
DAPI
RIME
Merge
Ctrl
KO
E
Ctrl
RIME KO
Relative IL-2 levels
<0.01
<0.01
KYSE70
KYSE520
F
Ctrl
RIME KO
RIME OE
IFN-γ
CD8
P5(50.71%)
P5(55.30%)
P5(43.41%)
G
% of IFN+ (of CD8+T cells)
<0.01
<0.01
Ctrl
RIME KO
RIME OE
H
Ctrl
RIME OE
Rel RIME levels
<0.01
<0.01
0.03
<0.01
<0.01
0.01
ns
<0.01
<0.01
<0.01
<0.01
ns
0.02
PD-L1
HLA-B
HLA-C
IDO-1
IL13
MICA
MICB
PD-L2
TGFB1
VEGFA
VISTA
VTCN1
CD47
I
Ctrl
RIME KO
Relative protein levels
<0.01
<0.01
PD-L1
IDO-1
KYSE70
Ctrl
RIME KO
Relative protein levels
<0.01
<0.05
PD-L1
IDO-1
KYSE520

Figure S2 *RIME* reduces sensitivity to the cytotoxicity of CD8$^+$ T cells by regulating immune checkpoint molecules

(A) The correlation of *RIME* expression level and tumour-infiltrating immune cells and tumour-killing activity was analysed based on The Cancer Genome Atlas (TCGA) transcriptome data and the CIBERSORT algorithm. The expression level of *RIME* was negatively correlated with tumour-infiltrating immune cells and tumour-killing activity. **(B)** Fluorescence in situ hybridization was performed to examine the subcellular localization of *RIME.* As shown, *RIME* was localized both in the nucleus and cytoplasm. Scale bar, 2 μm. **(C)** Sanger sequencing showed that *RIME* KO produced obvious overlapping peaks in the gene region near the *RIME* sgRNA, indicating that *RIME* KO sgRNA had strong cleavage activity on the target gene. Ctrl, control. **(D)** Fluorescence in situ hybridization showed that *RIME* KO efficiently inhibited *RIME* expression in ESCC cells. Scale bar, 2 μm. **(E)** ELISA assays showed that the IL-2 levels in the coculture medium were significantly increased in the *RIME* KO group. **(F-G)** Flow cytometry and statistical analysis showed that *RIME* KO significantly increased IFN-γ$^+$ CD8$^+$ T cells cocultured with ESCC cells. **(H)** qPCR analysis showed that *RIME* OE upregulated multiple immunosuppressive genes, among which *PD-L1* and *IDO-1* was the most significant. **(I)** Quantitative analysis of immunoblot assays showed that *RIME* KO decreased PD-L1 and IDO-1 expression levels.

Figure S3

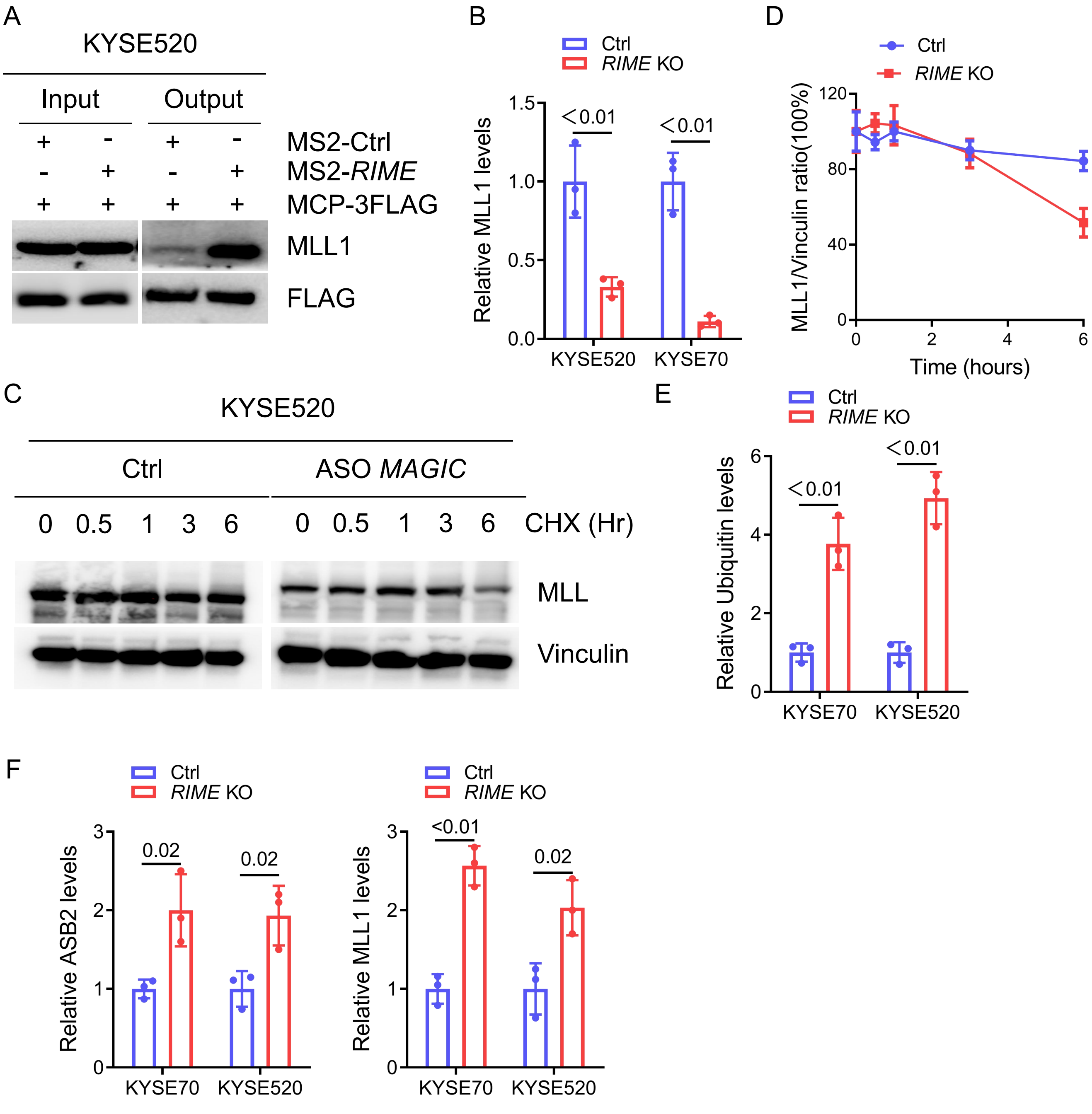

Figure S3 *RIME* binds and stabilizes MLL1

(A) The interaction of *RIME* and MLL1 *in vivo* was verified by MS2-tagged RNA affinity purification assays and immunoblot analysis in KYSE520 cells. **(B)** Quantitative analysis of immunoblot assays showed that *RIME* KO decreased MLL1 expression levels. **(C-D)** Immunoblot analysis and quantification of MLL1 protein levels in KYSE520 cells treated with CHX (100 μg/ml) for the indicated times. As shown, *RIME* KO significantly shortened the half-life of MLL1 in ESCC cells. **(E)** Quantitative analysis of immunoblot assays showed that *RIME* KO increased MLL1 ubiquitin levels. **(F)** Quantitative analysis of co-IP assays showed that *RIME* KO increased MLL1/ASB2 interaction.

Figure S4

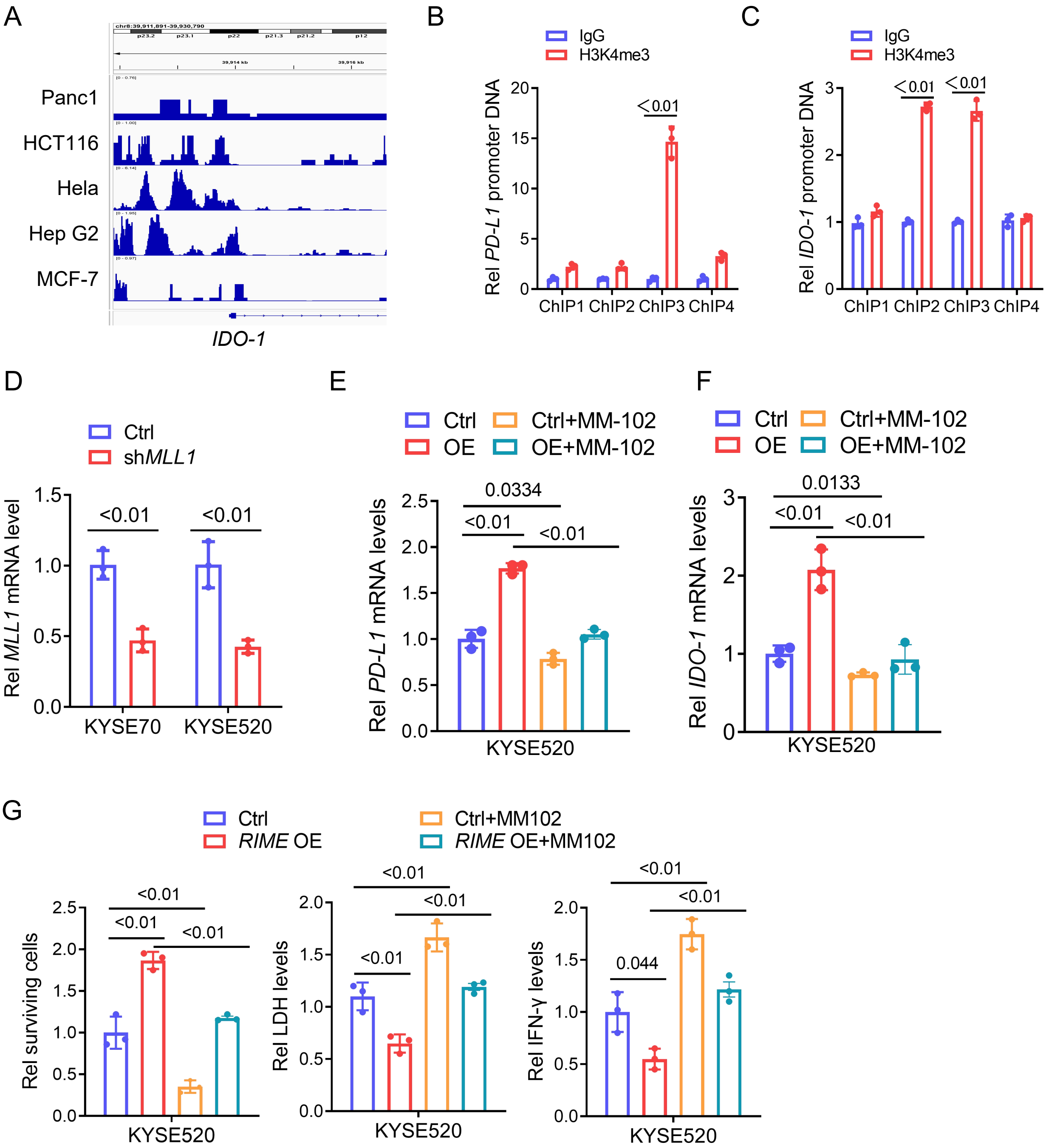

Figure S4 *RIME* regulates MLL1-H3K4me3-mediated PD-L1/IDO-1 expression

(A) ChIP-seq analysis showed that H3K4me3 levels were enriched in the *IDO-1* promoter regions. The ChIP-seq data was obtained from ENCODE database. **(B-C)** ChIP assays indicated that H3K4me3 marks were enriched in the *PD-L1* promoter region around -1000 to 0 bp, and in the *IDO-1* promoter region around -1500 to -500 bp. **(D)** qRT–PCR analysis verified that *MLL1* depletion by shRNA efficiently decreased *MLL1* mRNA levels in ESCC cells. **(E-F)** qRT–PCR analysis showed that the increased *PD-L1* and *IDO-1* expression levels induced by *RIME* overexpression were abolished by MLL1 inhibition (MM-102 treatment). **(G)** Crystal violet staining assay showed that MLL1 inhibition abolished the resistance of ESCC cells to cytotoxic CD8+ T cells induced by *RIME* overexpression. The decreased LDH and IFN-γ levels induced by *RIME* overexpression in the ESCC-T cell co-culture medium were also abolished by MLL1 inhibition.

Figure S5

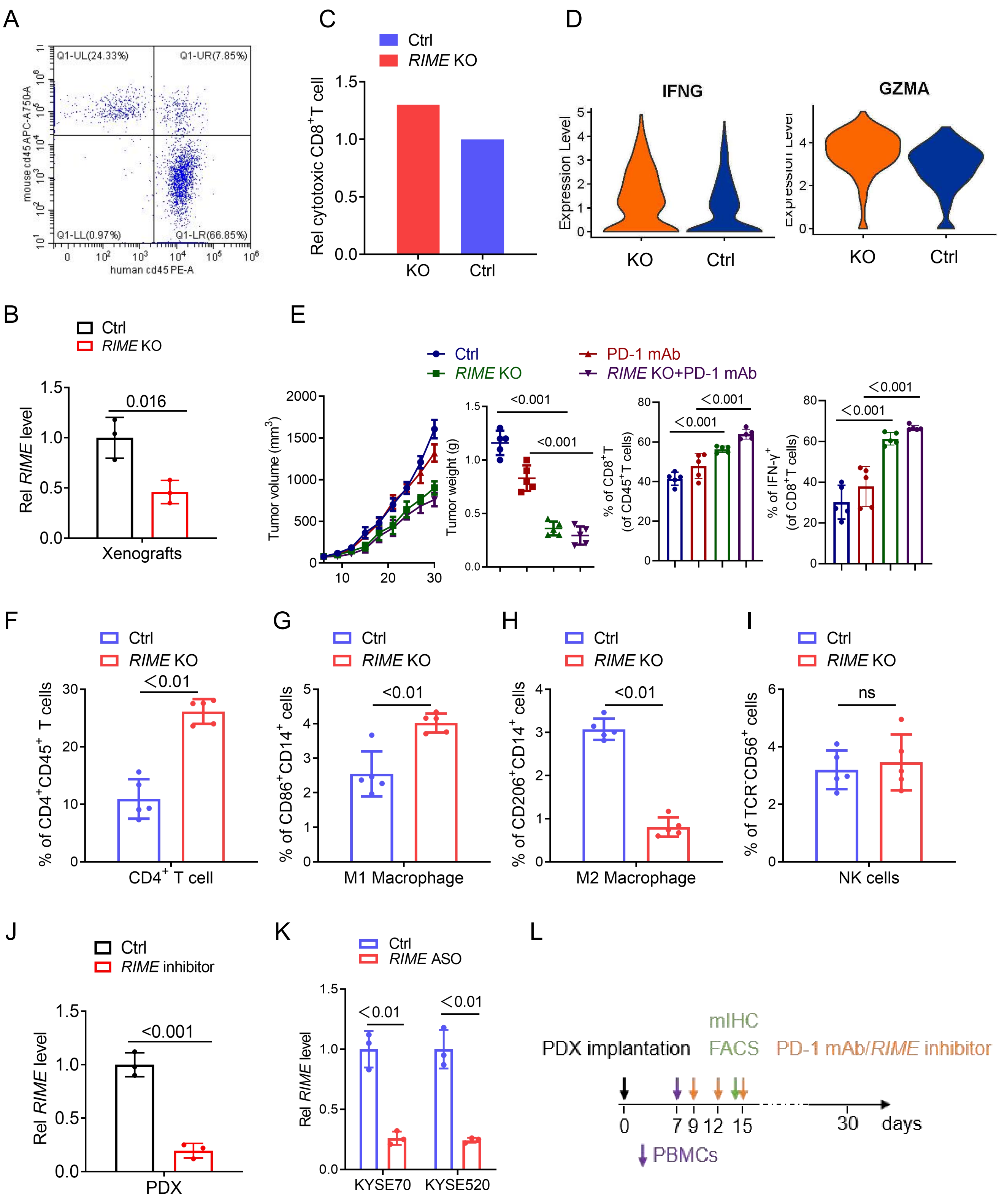
A
Q1-UL(24.33%)
Q1-UR(7.85%)
Q1-LL(0.97%)
Q1-LR(66.85%)
mouse cd45 APC-A750-A
human cd45 PE-A
B
Ctrl
RIME KO
Rel RIME level
0.016
Xenografts
C
Ctrl
RIME KO
Rel cytotoxic CD8+T cell
KO
Ctrl
D
IFNG
GZMA
Expression Level
KO
Ctrl
E
Ctrl
RIME KO
PD-1 mAb
RIME KO+PD-1 mAb
Tumor volume (mm3)
Tumor weight (g)
% of CD8+T (of CD45+T cells)
% of IFN-γ+ (of CD8+T cells)
<0.001
F
Ctrl
RIME KO
% of CD4+CD45+ T cells
<0.01
CD4+ T cell
G
Ctrl
RIME KO
% of CD86+CD14+ cells
<0.01
M1 Macrophage
H
Ctrl
RIME KO
% of CD206+CD14+ cells
<0.01
M2 Macrophage
I
Ctrl
RIME KO
% of TCR-CD56+ cells
ns
NK cells
J
Ctrl
RIME inhibitor
Rel RIME level
<0.001
PDX
K
Ctrl
RIME ASO
Rel RIME level
<0.01
KYSE70
KYSE520
L
mIHC
FACS
PDX implantation
PD-1 mAb/RIME inhibitor
0
7 9 12 15
30 days
PBMCs

Figure S5 *RIME* KO enhanced antitumour immunity in ESCC treatment

(A) Flow cytometry analysis of human-CD45$^+$ cells in hu-PBMC mouse peripheral blood. The proportion of human CD45$^+$ cells was more than 60%, implying that the huPBMC-NOG-CDX/PDX model was constructed successfully. **(B)** qRT–PCR analysis showed that *RIME* KO significantly suppressed *RIME* expression in ESCC xenografts. **(C)** ScRNA-seq analysis showed that *RIME* KO increased the number of cytotoxic CD8$^+$ T cells in the xenografts. **(D)** ScRNA-seq analysis showed that *RIME* KO upregulated the marker of cytotoxic CD8$^+$ T cells in the xenografts. Differentially expressed genes were identified using FindMarkers. **(E)** Flow cytometry analysis showed that *RIME* KO increased the proportion of CD8$^+$ T cells and IFN-γ$^+$ CD8$^+$ T cells in ESCC KYSE70 xenografts. **(F)** Flow cytometry analysis showed that *RIME* KO increased the proportion of CD4$^+$ T cells in ESCC xenografts. **(G)** Flow cytometry analysis showed that *RIME* KO increased the proportion of M1 macrophages in ESCC xenografts. **(H)** Flow cytometry analysis showed that *RIME* KO decreased the proportion of M2 macrophages in ESCC xenografts. **(I)** Flow cytometry analysis showed that *RIME* KO had no effect on the proportion of NK cells in ESCC xenografts.**(J)** qRT-PCR analysis showed that *RIME* inhibitor significantly decreased *RIME* level in tumour samples collected from Hu-PBMC-NOG-PDX mice. **(K)** qRT–PCR analysis showed that *RIME* ASO efficiently decreased *RIME* levels in ESCC cells. **(L)** Time points of administration and sampling in the Hu-PBMC-NOG-PDX mice.
