# Supplementary material for: Long noncoding RNA Regulating ImMune Escape regulates mixed lineage leukaemia protein‐1‐H3K4me3‐mediated immune escape in oesophageal squamous cell carcinoma
Source: Clin Transl Med. 2023 Sep 15;13(9):e1410. doi: 10.1002/ctm2.1410 (PMC10502462; doi:10.1002/ctm2.1410)
Supplement: Supplementary file 2 — Supporting Information [file CTM2-13-e1410-s001.docx]

**Table S1 The predicted off-target locus and primers used for PCR and Sanger sequencing**

Sequencing primers are indicated in red.

| Locus | l_seq | r_seq |
| --- | --- | --- |
| 1_AACAAAATGACTAAGCACCGNGG_chr6_106169673_+ | TGATCCTTCCCCCTCAGCTT | ATGGGGGAAATGGAGAGGGA |
| 2_AACAAAATGACTAAGCACCGNGG_chr5_115351516_+ | CGATTACCTCCCACCAGTCC | GCTCATCACTATCCAGGGCC |
| 3_AACAAAATGACTAAGCACCGNGG_chr3_131785712_- | CTTTGGGCCTTGCGGTTAAG | TTTCACCTTCCCCATGAGGC |
| 4_AACAAAATGACTAAGCACCGNGG_chr8_144390602_+ | CTGGCCCCGTTCTACTGATC | CGCAGACATCACCTCCAAGT |
| 5_AACAAAATGACTAAGCACCGNGG_chr6_126956733_- | AGTTGCTGTGTCAAAAACCAC | TACACCACCACACCCTGCTA |
| 6_CCCCCACCCGTCACCACCTTNGG_chr22_23458654_- | AAGGTGCCACGGTTAGACTG | CTTCTTCCCACACTGCCTCC |
| 7_CCCCCACCCGTCACCACCTTNGG_chr7_1817881_+ | ATGTGCCACGGTTAGACTGG | CTTCTTCCCACACTGCCTCC |
| 8_CCCCCACCCGTCACCACCTTNGG_chr18_61829057_- | TTCCTGCCTCCCCTTACAGT | CCATCCCAGCCTCCAGAGTA |
| 9_CCCCCACCCGTCACCACCTTNGG_chr4_187090934_- | CCCCTAGAACCCCAAGATGC | CGAGTTGTTACGAGCACCCT |
| 10_CCCCCACCCGTCACCACCTTNGG_chr2_214817363_- | CGGAAGGTGAATGAGGAGCA | TGCCTTGTTCATATTGGACTCA |

| **Table S2 *RIME* pull-down mass spectrum analysis 1** | | | | | |
| --- | --- | --- | --- | --- | --- |
| Score | Mass | Matches | Sequences | emPAI | Protein description |
| 39 | 130293 | 6 (4) | 2 (1) | 0.03 | Pyruvate carboxylase, mitochondrial OS=Homo sapiens OX=9606 GN=PC PE=1 SV=2 |
| 28 | 57173 | 1 (1) | 1 (1) | 0.06 | N-sulphoglucosamine sulphohydrolase OS=Homo sapiens OX=9606 GN=SGSH PE=1 SV=1 |
| 27 | 53592 | 3 (1) | 1 (1) | 0.06 | Keratin, type I cytoskeletal 12 OS=Homo sapiens OX=9606 GN=KRT12 PE=1 SV=1 |
| 27 | 39136 | 1 (1) | 1 (1) | 0.08 | Neurogenic differentiation factor 6 OS=Homo sapiens OX=9606 GN=NEUROD6 PE=2 SV=1 |
| 27 | 50413 | 3 (1) | 2 (1) | 0.07 | Putative protein FAM90A22P OS=Homo sapiens OX=9606 GN=FAM90A22P PE=5 SV=1 |
| 26 | 34375 | 3 (1) | 1 (1) | 0.1 | Heterogeneous nuclear ribonucleoprotein A1-like 2 OS=Homo sapiens OX=9606 GN=HNRNPA1L2 PE=2 SV=2 |
| 26 | 194423 | 2 (1) | 2 (1) | 0.02 | Intersectin-2 OS=Homo sapiens OX=9606 GN=ITSN2 PE=1 SV=3 |
| 25 | 201713 | 7 (2) | 3 (1) | 0.02 | Structure-specific endonuclease subunit SLX4 OS=Homo sapiens OX=9606 GN=SLX4 PE=1 SV=3 |
| 25 | 95070 | 10 (1) | 3 (1) | 0.03 | Lon protease homolog 2, peroxisomal OS=Homo sapiens OX=9606 GN=LONP2 PE=1 SV=1 |
| 23 | 56091 | 3 (2) | 1 (1) | 0.06 | KAT8 regulatory NSL complex subunit 2 OS=Homo sapiens OX=9606 GN=KANSL2 PE=1 SV=3 |
| 22 | 77180 | 2 (1) | 2 (1) | 0.04 | F-BAR and double SH3 domains protein 1 OS=Homo sapiens OX=9606 GN=FCHSD1 PE=1 SV=1 |
| 22 | 86630 | 1 (1) | 1 (1) | 0.04 | Protein spire homolog 1 OS=Homo sapiens OX=9606 GN=SPIRE1 PE=1 SV=3 |
| 22 | 275970 | 11 (1) | 5 (1) | 0.01 | Ankyrin repeat domain-containing protein 17 OS=Homo sapiens OX=9606 GN=ANKRD17 PE=1 SV=3 |
| 22 | 140055 | 14 (1) | 6 (1) | 0.02 | E3 ubiquitin-protein ligase DZIP3 OS=Homo sapiens OX=9606 GN=DZIP3 PE=1 SV=2 |
| 21 | 104498 | 4 (1) | 2 (1) | 0.03 | Interleukin-6 receptor subunit beta OS=Homo sapiens OX=9606 GN=IL6ST PE=1 SV=2 |
| 21 | 158508 | 4 (1) | 3 (1) | 0.02 | Condensin complex subunit 1 OS=Homo sapiens OX=9606 GN=NCAPD2 PE=1 SV=3 |
| 21 | 95002 | 2 (1) | 1 (1) | 0.03 | A-kinase anchor protein 2 OS=Homo sapiens OX=9606 GN=AKAP2 PE=1 SV=3 |
| 20 | 68880 | 4 (1) | 2 (1) | 0.05 | Kelch-like protein 20 OS=Homo sapiens OX=9606 GN=KLHL20 PE=1 SV=4 |
| 20 | 35796 | 1 (1) | 1 (1) | 0.09 | DTW domain-containing protein 1 OS=Homo sapiens OX=9606 GN=DTWD1 PE=1 SV=1 |
| 20 | 53054 | 2 (1) | 1 (1) | 0.06 | Ral-GDS-related protein OS=Homo sapiens OX=9606 GN=RGL4 PE=2 SV=1 |
| 20 | 66285 | 3 (1) | 2 (1) | 0.05 | Endoribonuclease ZC3H12A OS=Homo sapiens OX=9606 GN=ZC3H12A PE=1 SV=1 |
| 20 | 3842904 | 73 (1) | 55 (1) |  | Titin OS=Homo sapiens OX=9606 GN=TTN PE=1 SV=4 |
| 19 | 13507 | 4 (1) | 1 (1) | 0.25 | NADH dehydrogenase [ubiquinone] 1 alpha subcomplex subunit 5 OS=Homo sapiens OX=9606 GN=NDUFA5 PE=1 SV=3 |
| 19 | 228153 | 13 (1) | 8 (1) | 0.01 | Zinc finger C3H1 domain-containing protein OS=Homo sapiens OX=9606 GN=ZFC3H1 PE=1 SV=3 |
| 19 | 419230 | 6 (1) | 5 (1) | 0.01 | Spectrin beta chain, non-erythrocytic 5 OS=Homo sapiens OX=9606 GN=SPTBN5 PE=1 SV=2 |
| 19 | 436058 | 13 (1) | 9 (1) | 0.01 | Histone-lysine N-methyltransferase 2A OS=Homo sapiens OX=9606 GN=KMT2A PE=1 SV=5 |
| 18 | 72184 | 3 (1) | 2 (1) | 0.05 | RING finger protein 207 OS=Homo sapiens OX=9606 GN=RNF207 PE=1 SV=2 |
| 18 | 289467 | 12 (1) | 5 (1) | 0.01 | Zinc finger protein 462 OS=Homo sapiens OX=9606 GN=ZNF462 PE=1 SV=3 |
| 17 | 59950 | 2 (1) | 2 (1) | 0.05 | STE20/SPS1-related proline-alanine-rich protein kinase OS=Homo sapiens OX=9606 GN=STK39 PE=1 SV=3 |
| 17 | 55953 | 6 (1) | 3 (1) | 0.06 | Signal recognition particle 54 kDa protein OS=Homo sapiens OX=9606 GN=SRP54 PE=1 SV=1 |
| 17 | 24576 | 2 (1) | 1 (1) | 0.14 | DNA replication complex GINS protein PSF3 OS=Homo sapiens OX=9606 GN=GINS3 PE=1 SV=1 |
| 17 | 271665 | 4 (1) | 3 (1) | 0.01 | Microtubule-associated protein 1B OS=Homo sapiens OX=9606 GN=MAP1B PE=1 SV=2 |
| 17 | 53857 | 1 (1) | 1 (1) | 0.06 | Lipase member I OS=Homo sapiens OX=9606 GN=LIPI PE=1 SV=3 |
| 17 | 33718 | 2 (1) | 1 (1) | 0.1 | Uncharacterized protein C16orf86 OS=Homo sapiens OX=9606 GN=C16orf86 PE=2 SV=2 |
| 16 | 127932 | 3 (1) | 1 (1) | 0.03 | Integrin alpha-D OS=Homo sapiens OX=9606 GN=ITGAD PE=1 SV=2 |
| 16 | 127063 | 4 (1) | 2 (1) | 0.03 | Sodium-driven chloride bicarbonate exchanger OS=Homo sapiens OX=9606 GN=SLC4A10 PE=2 SV=1 |
| 15 | 192404 | 5 (1) | 3 (1) | 0.02 | Coiled-coil domain-containing protein 180 OS=Homo sapiens OX=9606 GN=CCDC180 PE=2 SV=2 |
| 15 | 27763 | 3 (1) | 1 (1) | 0.12 | Chymase OS=Homo sapiens OX=9606 GN=CMA1 PE=1 SV=1 |
| 15 | 54487 | 2 (1) | 1 (1) | 0.06 | Centrosomal protein of 55 kDa OS=Homo sapiens OX=9606 GN=CEP55 PE=1 SV=3 |
| 15 | 435957 | 7 (2) | 4 (1) | 0.01 | Ankyrin-2 OS=Homo sapiens OX=9606 GN=ANK2 PE=1 SV=4 |
| 15 | 134733 | 5 (1) | 2 (1) | 0.02 | von Willebrand factor A domain-containing protein 5B2 OS=Homo sapiens OX=9606 GN=VWA5B2 PE=2 SV=2 |
| 14 | 84686 | 1 (1) | 1 (1) | 0.04 | Forkhead box protein M1 OS=Homo sapiens OX=9606 GN=FOXM1 PE=1 SV=3 |
| 14 | 129015 | 3 (1) | 2 (1) | 0.03 | Transient receptor potential cation channel subfamily A member 1 OS=Homo sapiens OX=9606 GN=TRPA1 PE=1 SV=3 |
| 14 | 95205 | 8 (1) | 4 (1) | 0.03 | Tyrosine-protein kinase Fer OS=Homo sapiens OX=9606 GN=FER PE=1 SV=2 |
| 14 | 63638 | 2 (1) | 2 (1) | 0.05 | Copine-8 OS=Homo sapiens OX=9606 GN=CPNE8 PE=1 SV=2 |
| 14 | 150769 | 2 (1) | 1 (1) | 0.02 | Fanconi anemia group I protein OS=Homo sapiens OX=9606 GN=FANCI PE=1 SV=4 |
| 13 | 71066 | 7 (1) | 2 (1) | 0.05 | Very long-chain acyl-CoA synthetase OS=Homo sapiens OX=9606 GN=SLC27A2 PE=1 SV=2 |

Exponentially Modified Protein Abundance Index (emPAI) is an established method of estimating protein abundances from peptide counts in a single LC-MS/MS experiment. EmPAI is defined as 10^PAI^ minus one, where PAI (Protein Abundance Index) denotes the ratio of observed to observable peptides.

| **Table S3 *RIME* pull-down mass spectrum analysis 2** | | | | | | |
| --- | --- | --- | --- | --- | --- | --- |
| Score | Mass | Matches | Sequences | emPAI | Protein description |  |
| 35 | 97868 | 110 (44) | 4 (1) | 0.03 | Plakophilin-2 OS=Homo sapiens OX=9606 GN=PKP2 PE=1 SV=2 |  |
| 35 | 143804 | 7 (1) | 5 (1) | 0.02 | Peroxisome biogenesis factor 1 OS=Homo sapiens OX=9606 GN=PEX1 PE=1 SV=1 |  |
| 33 | 29331 | 1 (1) | 1 (1) | 0.11 | Keratinocyte-associated transmembrane protein 2 OS=Homo sapiens OX=9606 GN=KCT2 PE=1 SV=2 |  |
| 32 | 57985 | 4 (1) | 3 (1) | 0.06 | Keratin, type II cuticular Hb2 OS=Homo sapiens OX=9606 GN=KRT82 PE=3 SV=3 |  |
| 31 | 77180 | 2 (1) | 2 (1) | 0.04 | F-BAR and double SH3 domains protein 1 OS=Homo sapiens OX=9606 GN=FCHSD1 PE=1 SV=1 |  |
| 28 | 183280 | 9 (1) | 4 (1) | 0.02 | Mitogen-activated protein kinase kinase kinase 4 OS=Homo sapiens OX=9606 GN=MAP3K4 PE=1 SV=2 |  |
| 28 | 158792 | 7 (1) | 4 (1) | 0.02 | Histone-lysine N-methyltransferase 2A OS=Homo sapiens OX=9606 GN=KMT2A PE=1 SV=5 |  |
| 28 | 220601 | 8 (1) | 5 (1) | 0.01 | Probable helicase with zinc finger domain OS=Homo sapiens OX=9606 GN=HELZ PE=1 SV=2 |  |
| 27 | 102268 | 2 (1) | 1 (1) | 0.03 | Trafficking kinesin-binding protein 2 OS=Homo sapiens OX=9606 GN=TRAK2 PE=1 SV=2 |  |
| 26 | 178476 | 14 (1) | 4 (1) | 0.02 | ATP-binding cassette sub-family C member 8 OS=Homo sapiens OX=9606 GN=ABCC8 PE=1 SV=6 |  |
| 25 | 91376 | 32 (6) | 3 (1) | 0.04 | Ras GTPase-activating protein 4B OS=Homo sapiens OX=9606 GN=RASA4B PE=3 SV=2 |  |
| 25 | 107895 | 35 (3) | 3 (1) | 0.03 | Apical junction component 1 homolog OS=Homo sapiens OX=9606 GN=AJM1 PE=3 SV=1 |  |
| 25 | 130375 | 10 (1) | 4 (1) | 0.03 | Coiled-coil domain-containing protein 40 OS=Homo sapiens OX=9606 GN=CCDC40 PE=2 SV=2 |  |
| 24 | 229231 | 13 (2) | 8 (1) | 0.01 | Protein Daple OS=Homo sapiens OX=9606 GN=CCDC88C PE=1 SV=3 |  |
| 24 | 136764 | 13 (1) | 1 (1) | 0.02 | PAN2-PAN3 deadenylation complex catalytic subunit PAN2 OS=Homo sapiens OX=9606 GN=PAN2 PE=1 SV=3 |  |
| 23 | 139469 | 5 (1) | 3 (1) | 0.02 | A disintegrin and metalloproteinase with thrombospondin motifs 18 OS=Homo sapiens OX=9606 GN=ADAMTS18 PE=1 SV=3 |  |
| 23 | 99398 | 5 (1) | 2 (1) | 0.03 | SKI/DACH domain-containing protein 1 OS=Homo sapiens OX=9606 GN=SKIDA1 PE=1 SV=2 |  |
| 22 | 365271 | 19 (1) | 9 (1) | 0.01 | Cilia- and flagella-associated protein 47 OS=Homo sapiens OX=9606 GN=CFAP47 PE=2 SV=5 |  |
| 21 | 59286 | 6 (1) | 3 (1) | 0.06 | Histone acetyltransferase KAT5 OS=Homo sapiens OX=9606 GN=KAT5 PE=1 SV=2 |  |
| 21 | 104498 | 6 (1) | 2 (1) | 0.03 | Interleukin-6 receptor subunit beta OS=Homo sapiens OX=9606 GN=IL6ST PE=1 SV=2 |  |
| 21 | 103752 | 3 (2) | 2 (1) | 0.03 | Probable 2-oxoglutarate dehydrogenase E1 component DHKTD1, mitochondrial OS=Homo sapiens OX=9606 GN=DHTKD1 PE=1 SV=2 |  |
| 20 | 73013 | 30 (1) | 2 (1) | 0.04 | Myotubularin-related protein 14 OS=Homo sapiens OX=9606 GN=MTMR14 PE=1 SV=2 |  |
| 20 | 62383 | 43 (1) | 2 (1) | 0.05 | Acyl-coenzyme A oxidase-like protein OS=Homo sapiens OX=9606 GN=ACOXL PE=2 SV=3 |  |
| 20 | 194739 | 5 (1) | 2 (1) | 0.02 | Trinucleotide repeat-containing gene 6B protein OS=Homo sapiens OX=9606 GN=TNRC6B PE=1 SV=4 |  |
| 20 | 162036 | 7 (2) | 4 (1) | 0.02 | Cleavage and polyadenylation specificity factor subunit 1 OS=Homo sapiens OX=9606 GN=CPSF1 PE=1 SV=2 |  |
| 19 | 225780 | 14 (1) | 6 (1) | 0.01 | Uncharacterized protein C2orf16 OS=Homo sapiens OX=9606 GN=C2orf16 PE=2 SV=3 |  |
| 19 | 149713 | 24 (1) | 3 (1) | 0.02 | Protein FAM205A OS=Homo sapiens OX=9606 GN=FAM205A PE=2 SV=4 |  |
| 18 | 177682 | 9 (1) | 5 (1) | 0.02 | Eukaryotic translation initiation factor 4 gamma 3 OS=Homo sapiens OX=9606 GN=EIF4G3 PE=1 SV=2 |  |
| 18 | 62074 | 2 (2) | 1 (1) | 0.05 | Tyrosine-protein kinase TXK OS=Homo sapiens OX=9606 GN=TXK PE=1 SV=3 |  |
| 17 | 110117 | 1 (1) | 1 (1) | 0.03 | Disks large-associated protein 1 OS=Homo sapiens OX=9606 GN=DLGAP1 PE=1 SV=1 |  |
| 16 | 136602 | 4 (1) | 1 (1) | 0.02 | Transcriptional activator MN1 OS=Homo sapiens OX=9606 GN=MN1 PE=1 SV=3 |  |
| 14 | 360698 | 13 (1) | 11 (1) | 0.01 | Proliferation marker protein Ki-67 OS=Homo sapiens OX=9606 GN=MKI67 PE=1 SV=2 |  |
| 14 | 105592 | 4 (1) | 3 (1) | 0.03 | Histone-lysine N-methyltransferase PRDM9 OS=Homo sapiens OX=9606 GN=PRDM9 PE=1 SV=2 |  |
| 14 | 53666 | 2 (1) | 1 (1) | 0.06 | Interferon regulatory factor 6 OS=Homo sapiens OX=9606 GN=IRF6 PE=1 SV=1 |  |
| 14 | 216593 | 4 (1) | 4 (1) | 0.02 | Girdin OS=Homo sapiens OX=9606 GN=CCDC88A PE=1 SV=2 |  |
| 14 | 38935 | 3 (1) | 3 (1) | 0.08 | Transcription factor EC OS=Homo sapiens OX=9606 GN=TFEC PE=1 SV=1 |  |
| 14 | 20850 | 4 (1) | 3 (1) | 0.16 | Histone H1.0 OS=Homo sapiens OX=9606 GN=H1F0 PE=1 SV=3 |  |
| 14 | 280157 | 18 (1) | 7 (1) | 0.01 | Filamin-B OS=Homo sapiens OX=9606 GN=FLNB PE=1 SV=2 |  |
| 13 | 100664 | 27 (1) | 3 (1) | 0.03 | Androgen receptor OS=Homo sapiens OX=9606 GN=AR PE=1 SV=3 |  |
| 13 | 96934 | 7 (1) | 4 (1) | 0.03 | Mismatch repair endonuclease PMS2 OS=Homo sapiens OX=9606 GN=PMS2 PE=1 SV=2 |  |

**.**

**Table S4. Correlation between *RIME* and clinicopathological characteristics in 58 ESCC patients**

| Characteristic | Low *RIME* (n=15) | High *RIME*(n=43) | *P* value |
| --- | --- | --- | --- |
| Age |  |  |  |
| ≥60 | 8(53.3%) | 17(39.5%) | 0.3808 |
| <60 | 7(46.7%) | 26(60.5%) |  |
| Gender |  |  |  |
| Male | 4(26.7%) | 6(14.0%) | 0.2651 |
| Female | 11(73.3%) | 37(86.0%) |  |
| MSI status |  |  |  |
| MSS | 15(100.0%) | 43(100.0%) | / |
| MSI-High | 0(0.0%) | 0(0.0%) |  |
| PD-L1 TC |  |  |  |
| <1 | 11(73.3%) | 36(83.7%) | 0.4502 |
| ≥1 | 4(26.7%) | 7(16.3%) |  |
| PD-L1 IC |  |  |  |
| <1 | 12(80.0%) | 30(70.0%) | 0.5221 |
| ≥1 | 3(20.0%) | 13(30.0%) |  |
| TMB Muts |  |  |  |
| <10 | 13(86.7%) | 39(90.7%) | 0.4917 |
| ≥10 | 2(13.3%) | 14(9.3%) |  |
| The *P* value was determined by a Fisher’s exact test. | | | |

**Table S5 The sequences of primers used for q-RT-PCR assay in this study**

| Gene name | Sequence |
| --- | --- |
| *RIME (LINC02096)* | F: TTCTGAGGCCCACGATAAGC  R: TGGAGATGTGAGTTGCTGCC |
| *β-Actin* | F: TGGATCAGCAAGCAGGAGTA  R: TCGGCCACATTGTGAACTTT |
| *PD-L1* | F: TGGCATTTGCTGAACGCATTT  R: TGCAGCCAGGTCTAATTGTTTT |
| *IDO-1* | F: GCCAGCTTCGAGAAAGAGTTG  R: ATCCCAGAACTAGACGTGCAA |
| *MLL1* | F: GGATCCACAACTCCAGGCAA  R: ATTTGGAATGGACCCAGCGA |
| *CD68* | F:GGAAATGCCACGGTTCATCCA  R:TGGGGTTCAGTACAGAGATGC |

**Table S6 The sequences of sgRNA and RNA inhibitor used in this study**

| Name | Sequence |
| --- | --- |
| *RIME* sgRNA1 | CCCCCACCCGTCACCACCTT |
| *RIME* sgRNA2 | AACAAAATGACTAAGCACCG |
| *MLL1* shRNA | GCATGTGAACCAGCTCCTT |
| *RIME* inhibitor | AGCGCACCGAAAGGAACCAA |

**Table S7 Key resources information**

| REAGENT or RESOURCE | SOURCE | IDENTIFIER |
| --- | --- | --- |
| Antibodies | | |
| Anti-ubiquitin | Cell Signaling Technology | Cat# 3933 |
| Anti-MLL1 | Cell Signaling Technology | Cat# 14689 |
| Anti-FLAG tag | Cell Signaling Technology | Cat# 8146 |
| Anti-Vinculin | Cell Signaling Technology | Cat# 13901 |
| Anti-CD68 | ZSGB-BIO | Cat# ZM-0060 |
| Anti-CD8 | Abcam | Cat# 217344 |
| Anti-Granzyme B | Abcam | Cat# ab208586 |
| Anti-PD-L1 | Abcam | Cat# ab228415 |
| Anti-IDO-1 | Abcam | Cat# 211017 |
| Anti-CD14 microbeads | Miltenyi Biotec | Cat# 130-050-201 |
| Anti-PDL1-BV421 | Biolegend | Cat# 329713 |
| Anti-IDO1-Alexa 647 | BD | Cat# 566648 |
| Anti-TCR α/β-PerCP/Cyanine5.5 | Biolegend | Cat# 306724 |
| Anti-CD8-APC | Biolegend | Cat# 344722 |
| Anti-CD45-PE | Biolegend | Cat# 368510 |
| Anti-IFN-ɣ- BV421 | Biolegend | Cat# 502532 |
| Alexa Fluor® 488-labelled secondary antibody | Invitrogen | Cat# A11008 |
| Chemicals | | |
| RPMI Medium 1640 basic (1X) | GIBCO | Cat# C11875500BT |
| Fetal bovine serum | Wisent Inc | Cat# 086-150 |
| Penicillin-Streptomycin | GIBCO | Cat# 15140163 |
| Lipofectamine 3000 | Invitrogen | Cat# L3000015 |
| Puromycin | Solarbio | Cat# P8230 |
| Trizol reagent | Life technologies | Cat# 15596018 |
| TritonTM X-100 | Sigma-Aldrich | Cat# C111549 |
| PMSF | Roche | Cat# 10837091001 |
| Immobilized Trypsin | Promega | Cat# V9012 |
| MG-132 | Selleck Chemicals | Cat# S2619 |
| CHX | Selleck Chemicals | Cat# S7418 |
| Zombie Aqua Fixable Viability Kit | Biolegend | Cat# 423102 |
| BD Cytofix/Cytoperm™ Fixation and Permeabilization Solution | BD | Cat# 554714 |
| Critical commercial assays | | |
| ProLong™ Gold Antifade Mountant with DAPI | Invitrogen | Cat# P36931 |
| Fluorescent *in Situ* Hybridization Kit | RiboBio | Cat# C10910 |
| MEGAscript™ T7 Transcription Kit | Invitrogen | Cat# AM1334 |
| Pierce RNA 3' End Desthiobiotinylation Kit | Thermo Scientific | Cat# 20163 |
| Pierce Magnetic RNA-Protein Pull-Down Kit | Thermo Scientific | Cat# 2016 |
| Magna RBP Immunoprecipitation Kit | Millipore | Cat# 17-700 |
| GoTaq qPCR Master Mix Kit | Promega | Cat# A6002 |
| BCA assay kit | Thermo Scientific | Cat# 23227 |
| Pierce Protein A/G Magnetic Beads | Thermo Scientific | Cat# 88803 |
| Duolink PLA Kit | Sigma-Aldrich | Cat# DUO92101 |
| Experimental models: Cell lines | | |
| KYSE30, KYSE150, KYSE70, KYSE520 | German Cell Culture Collection | Cat#: ACC351, ACC357, ACC363, ACC 379, ACC374, ACC371 |
| TE-9 and TE-11 cell lines | RIKEN BioResource Research Center | Cat#: RCB1988, RCB2100 |
|  | | |
